# The Actin Nucleator Cobl Is Controlled by Calcium and Calmodulin

**DOI:** 10.1371/journal.pbio.1002233

**Published:** 2015-09-03

**Authors:** Wenya Hou, Maryam Izadi, Sabine Nemitz, Natja Haag, Michael M. Kessels, Britta Qualmann

**Affiliations:** Institute of Biochemistry I, Jena University Hospital/Friedrich-Schiller-University Jena, Jena, Germany; University of Glasgow, UNITED KINGDOM

## Abstract

Actin nucleation triggers the formation of new actin filaments and has the power to shape cells but requires tight control in order to bring about proper morphologies. The regulation of the members of the novel class of WASP Homology 2 (WH2) domain-based actin nucleators, however, thus far has largely remained elusive. Our study reveals signal cascades and mechanisms regulating Cordon-Bleu (Cobl). Cobl plays some, albeit not fully understood, role in early arborization of neurons and nucleates actin by a mechanism that requires a combination of all three of its actin monomer–binding WH2 domains. Our experiments reveal that Cobl is regulated by Ca^2+^ and multiple, direct associations of the Ca^2+^ sensor Calmodulin (CaM). Overexpression analyses and rescue experiments of Cobl loss-of-function phenotypes with Cobl mutants in primary neurons and in tissue slices demonstrated the importance of CaM binding for Cobl’s functions. Cobl-induced dendritic branch initiation was preceded by Ca^2+^ signals and coincided with local F-actin and CaM accumulations. CaM inhibitor studies showed that Cobl-mediated branching is strictly dependent on CaM activity. Mechanistic studies revealed that Ca^2+^/CaM modulates Cobl’s actin binding properties and furthermore promotes Cobl’s previously identified interactions with the membrane-shaping F-BAR protein syndapin I, which accumulated with Cobl at nascent dendritic protrusion sites. The findings of our study demonstrate a direct regulation of an actin nucleator by Ca^2+^/CaM and reveal that the Ca^2+^/CaM-controlled molecular mechanisms we discovered are crucial for Cobl’s cellular functions. By unveiling the means of Cobl regulation and the mechanisms, by which Ca^2+^/CaM signals directly converge on a cellular effector promoting actin filament formation, our work furthermore sheds light on how local Ca^2+^ signals steer and power branch initiation during early arborization of nerve cells—a key process in neuronal network formation.

## Abstract 2

The calcium sensor calmodulin directly regulates the actin filament-promoting factor Cobl to help shape the complex architecture of neurons underlying neuronal network formation.

## Author Summary

The organization and the formation of new actin filaments by polymerization of actin monomers has the power to shape cells. The rate-limiting step in actin polymerization is “nucleation”—a process during which the first actin monomers are assembled with the help of actin nucleators. This nucleation step requires tight temporal and spatial control in order to achieve proper cell morphologies. Here, we analyse signaling cascades and mechanisms regulating the actin nucleator Cobl, which is crucial for the formation of dendritic arbors of nerve cells—a key process in neuronal network formation. We show that the calcium (Ca^2+^)-binding signaling component calmodulin (CaM) binds to Cobl and regulates its functions. Using 3-D time-lapse analyses of developing neurons, we visualized how Cobl works. We observed local accumulation of CaM, Cobl, actin, and syndapin I—a membrane-shaping protein—at dendritic branch initiation sites. We find that Ca^2+^/CaM modulates Cobl’s actin-binding properties and promotes its interactions with syndapin I, which then serves as a membrane anchor for Cobl. In summary, we i) show a direct regulation of the actin nucleator Cobl by Ca^2+^/CaM, ii) demonstrate that the molecular mechanisms we discovered are crucial for shaping nerve cells, and iii) underscore how local Ca^2+^ signals steer and power branch initiation during early arborization of neurons.

## Introduction

Metazoan life critically relies on the formation, organization, and dynamics of actin filaments, which are, for example, crucial for shaping and movement of membranes and entire cells. The polar and extremely arborized morphologies that neurons develop during pre- and postnatal brain development are a prerequisite for signal processing in neuronal networks. Their development seems to be promoted by cytoskeletal structures and local calcium signals. These Ca^2+^ signals are mediated by N-methyl-D-aspartic acid (NMDA)-type glutamate receptors, voltage-gated calcium channels, and ryanodine receptors [1–3] and seem to be sensed by the Ca^2+^-binding protein calmodulin (CaM; M19312.1; GI:203255), because CaM kinases (CaMKs) downstream of CaM were observed to be involved in dendritogenesis [4,5].

Prime effector machinery that may power early neuromorphogenesis would be proteins with the ability to trigger the formation of new actin filaments in a spatially and locally well-controlled manner. The well-established actin filament-promoting components, i.e., the Arp2/3 complex and Formins, are controlled by Rho-type GTPases [6–9]. Actin nucleators that respond to Ca^2+^/CaM signals directly are not known. With Cobl (NM_172496.3; GI:162135965) and JMY (NM_021310.3; GI:326633181), two members of the novel class of Wiskott-Aldrich syndrome protein (WASP) Homology 2 (WH2) domain-based actin nucleators [10,11] have been implicated in the development of early neuronal morphology in different ways [12,13]. Cobl nucleates actin filaments by a mechanism that requires a combination of all three of its C-terminal actin monomer-binding WH2 domains [12]. In vitro, a WH2 domain–containing C-terminal fragment of human Cobl additionally increased actin dynamics by severing filaments [14]. Functional studies in neurons showed that Cobl plays some, albeit not fully understood, role in early arborization of hippocampal neurons and of cerebellar Purkinje cells [12,15]. These functions rely on associations of Cobl with the F-BAR protein syndapin I (AF104402.1; GI:4324451) and the F-actin-binding protein Abp1 (NM_001146308.1; GI:226423870) as well as on all three C-terminal WH2 domains of Cobl [12,15,16].

Here, we reveal a signaling pathway that controls the molecular functions of Cobl and identify a direct link between Ca^2+^/CaM signaling and the early morphogenesis of neurons. We demonstrate that Cobl is a direct target of Ca^2+^-activated CaM and that Ca^2+^/CaM activity and CaM interactions are crucial for the Cobl-mediated development of dendritic arbors. Ca^2+^/CaM modulates Cobl’s actin binding properties. Furthermore, we show that CaM association promotes Cobl’s interactions with syndapin I and that Cobl, CaM, F-actin, and syndapin I accumulate at branch initiation sites.

Taken together, our study addresses the means of Cobl regulation and reveals several molecular mechanisms that enable the Ca^2+^/CaM signaling pathway to steer Cobl. With Cobl, we identified an actin filament–promoting effector by which local Ca^2+^/CaM-signaling directly powers cellular morphogenesis during early neuronal network formation.

## Results

### Dendritic Protrusions Initiate from Cobl and F-actin Accumulations

**Fig 1:**
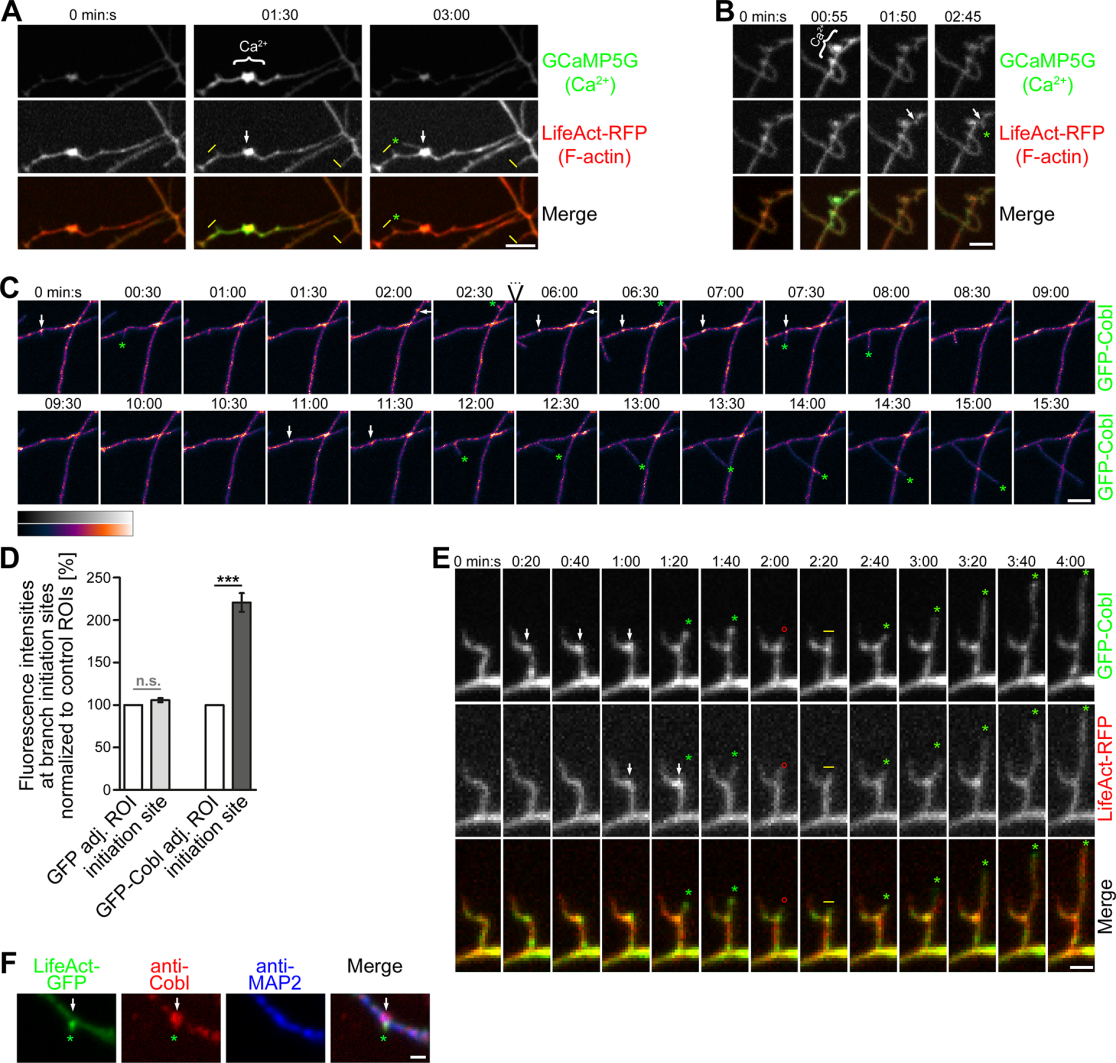
Dendrite branching coincides with local Ca^2+^ signals and Cobl accumulation leading to bursts of F-actin formation at the base of initiating dendritic protrusions. (**A,B**) Individual frames (maximum intensity projections [MIPs]) of 3-D-time-lapse recordings of dendritic, locally restricted Ca^2+^ signals in developing primary hippocampal neurons visualized by GCaMP5G (marked by}) and F-actin accumulation visualized using LifeAct-RFP (transfection, days in vitro (DIV)6; imaging, DIV7). Local increase of intracellular Ca^2+^ levels correlates with subsequent protrusion (arrows mark initiation sites) formation (marked by green *) from a previously existing F-actin–rich site (**A**) and from a site that newly acquired F-actin accumulation after a Ca^2+^ signal (**B**). Static protrusions are marked by yellow I. Bars in **A** and **B**, 5 μm. (**C**) Example images (MIPs) of 3-D-time-lapse recording (shown are 0–2:30 and 6:00–15:30 min:s) of GFP-Cobl reveal that Cobl is dynamically enriched at distinct sites within dendrites in immature neurons undergoing dendritogenesis. Initiation of dynamic, dendritic protrusions (marked by green *) often is preceded by Cobl accumulation (white arrows). Retraction events are marked by red ° and static phases with yellow I. Dendrite branch induction is a dynamic process with often several protrusive attempts until a dendritic branch is firmly established and strongly elongated. Three consecutive initiations of a protrusion from the same site are shown. Data (see S1 Fig for original data of the GFP channel) are shown in a color-coded manner (heat map purple to white, see legend for color coding). Bar, 5 μm. Please also see S1 Movie. (**D**) Quantitative analyses of GFP-Cobl enrichment at dendritic protrusion initiation sites 30 s prior to protrusion initiation in comparison to adjacent control regions of interest (ROIs). Errors represent standard error of the mean (SEM). Statistical significances, one-way ANOVA with Tukey’s post test. GFP-Cobl, *n* = 25; GFP, *n* = 18. ***p < 0.001. For underlying data, see S1 Data. (**E**) Simultaneous recordings of GFP-Cobl and LifeAct-RFP show that initiation of dendritic protrusions coincides with Cobl accumulation followed by F-actin buildup at the dendritic base (arrows). Labeling as in **C**. Bar, 2 μm. Please also see S2 Movie. (**F**) Anti-Cobl and anti-MAP2-stained hippocampal neuron at DIV6 transfected with LifeAct-GFP to mark putative sites of dendritic branch induction show colocalization of Cobl with F-actin at actin-rich sites protruding from the dendrites (arrow). Bar, 1 μm.

The extremely arborized morphologies that neurons develop during their early morphogenesis are a prerequisite for the formation of all neuronal networks. By some, yet unknown means, the underlying reorganization of cell shape involves Cobl, a protein that has the ability to promote the formation of new actin filaments [12,14]. Dendritic arborization has furthermore been suggested to be controlled by local Ca^2+^ influx. Indeed, 3-D-time-lapse calcium imaging in developing hippocampal neurons showed that protrusions, which formed in areas marked by transient Ca^2+^ influx, were originating from sites enriched for F-actin. These F-actin-rich sites either already existed prior to the calcium signal (Fig 1A) or F-actin accumulated after a calcium pulse (Fig 1B).

Analyzing the distribution and dynamics of the actin nucleator Cobl in developing neurons, we observed that the formation of protrusions from dendritic structures coincided with Cobl enrichment. Branching was a dynamic process with branches being initiated, shrinking back to the mother dendrite and being reinitiated until they were firmly established and grew out. Cobl accumulated in spatially restricted dendritic sites prior to the induction of almost all branching events (Fig 1C, arrows; S1 Fig; S1 Movie). Quantitative analyses showed that Cobl was highly enriched at branch initiation sites 30 s before initiation of protrusion. The intensity of GFP-Cobl at such sites was more than twice as high as at adjacent control region of interest (ROI), whereas GFP showed no intensity differences when control ROIs were compared to branch initiation sites (Fig 1D).

Dual imaging of GFP-Cobl and LifeAct-RFP revealed that Cobl accumulations and dendritic branch inductions were accompanied by local F-actin formation. Interestingly, the signals of both Cobl and F-actin were particularly high at the base of initiated branches. Furthermore, we observed that the maximum of Cobl accumulation hereby usually preceded that of F-actin accumulation (Fig 1E; S2 Movie).

In line with the live imaging data, immunostainings of dendrites of developing neurons showed that sites marked by increased F-actin were often marked by protrusive morphology and displayed accumulations of endogenous Cobl (Fig 1F).

### Cobl Interacts with CaM

**Fig 2:**
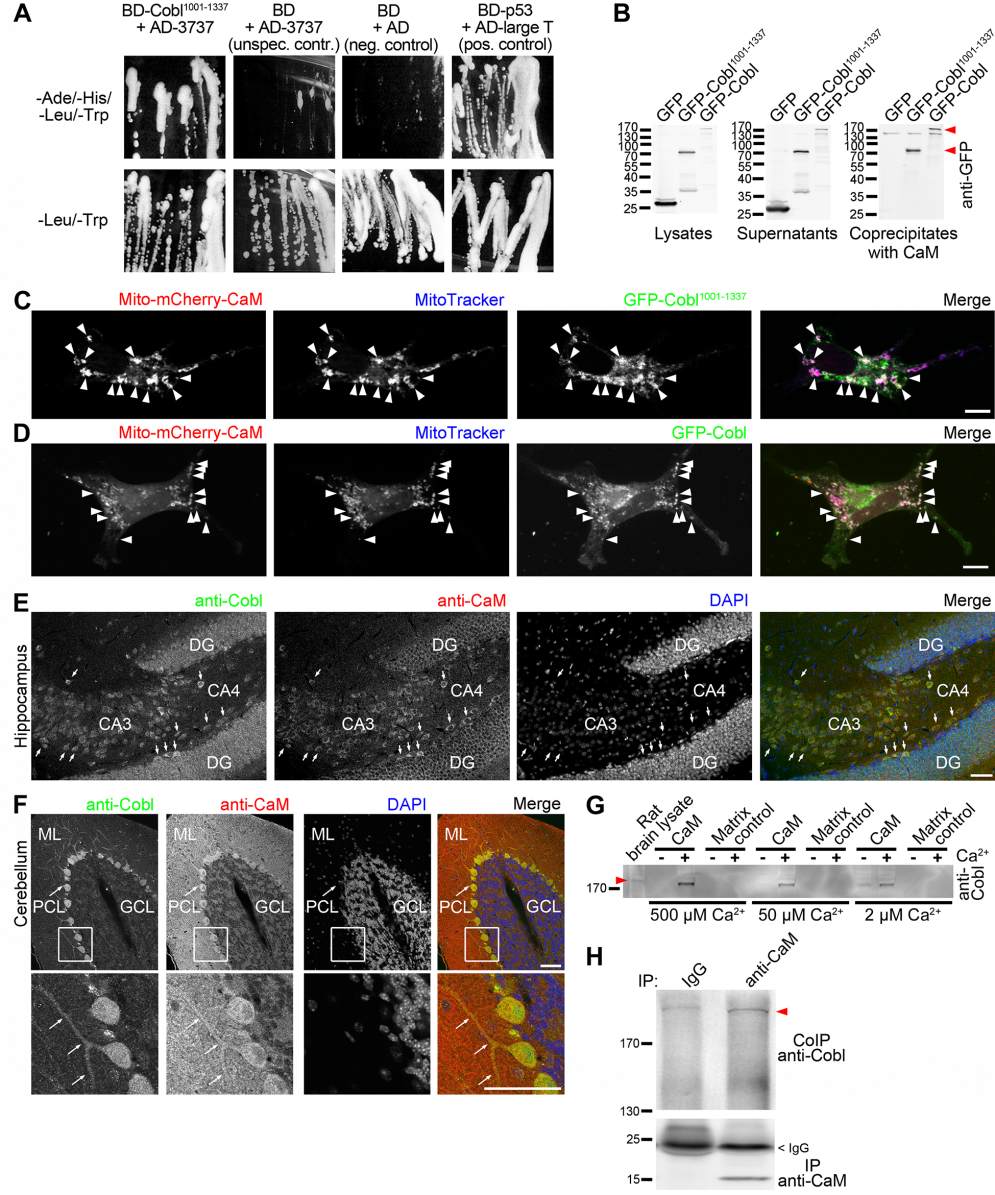
Cobl is a calmodulin-binding protein. (**A**) Yeast-2-hybrid verification of hit#3737 encoding CaM^∆1–32^ using BD-Cobl^1001–1337^ as bait and isolated and retransformed plasmid AD-3737 as prey. Reporter gene activities manifest in growth on quadruple dropout media (-Ade/-His/-Leu/-Trp). Growth on-Leu/-Trp medium confirms coexpression of both plasmids. (**B**) Coprecipitation analyses with immobilized CaM and GFP-Cobl^1001–1337^ and GFP-Cobl (arrowheads mark bands) expressed in HEK293 cells verify that full-length CaM (under 500 μM Ca^2+^) binds to Cobl. (**C,D**) Visualization of the Cobl/CaM interaction in intact COS-7 cells by recruitment of GFP-Cobl^1001–1337^ (**C**) and GFP-Cobl (**D**) to mitochondrially anchored mCherry-CaM (Mito-mCherry-CaM). Examples of successful CaM/Cobl complex reconstitution are marked with arrowheads. Bars in **C and D**, 10 μm. For establishment of mitochondrial targeting of Mito-mCherry-CaM and GFP-control of the Cobl recruitment and specificity control experiments see S2 Fig. (**E,F**) Anti-Cobl and anti-CaM immunostaining of parasagittal sections of the hippocampus (**E)** and cerebellum (**F**) at high magnifications demonstrate that Cobl and CaM are enriched in the same cells in the CA3 and CA4 regions (**E**; examples marked with short arrows) and in Purkinje cells of the cerebellum including their dendritic arbor (**F**; long arrows). Lower panels, enlargements of boxed areas. DG, dentate gyrus; ML, Molecular Layer; PCL, Purkinje Cell Layer; GCL, Granule Cell Layer. Bars, 50 μm. (**G**) Coprecipitation analyses with immobilized CaM and brain lysates demonstrating that the Cobl/CaM interaction is Ca^2+^dependent and that CaM complexes with endogenous Cobl (arrowhead marks bands) are formed at all Ca^2+^ concentrations tested. (**H**) Coimmunoprecipitation of endogenous Cobl (arrowhead marks band) with anti-CaM antibodies. For characterization of anti-CaM antibodies in immunoprecipitations and immunoblotting please see S3 Fig).

In contrast to other cytoskeletal effectors that give rise to new actin filaments, Cobl has no obvious Rho-type GTPase-binding domains or other regulatory modules that may help to control this powerful cellular machine. We therefore conducted a yeast-2-hybrid screen with BD-Cobl^1001–1337^ as bait to identify cellular mechanisms that may be responsible for the observed local control of Cobl during branch initiation. Hit#3737 encoded for AD-CaM^∆1–32^. Retransformed AD-3737-plasmid led to robust reporter gene activity when combined with BD-Cobl^1001–1337^ (Fig 2A).

Coprecipitations confirmed that GFP-Cobl^1001–1337^ and GFP-Cobl specifically bound to immobilized CaM (Fig 2B).

Successful and specific reconstitutions of GFP-Cobl/CaM and GFP-Cobl^1001–1337^/CaM complexes at defined sites in intact COS-7 cells using mitochondrially targeted CaM (Mito-mCherry-CaM) demonstrated the in vivo relevance of the Cobl/CaM interactions (Fig 2C and 2D; S2 Fig).

Immunohistological examinations showed that Cobl and CaM display overlapping expression. Cells with pronounced Cobl expression in the hippocampus were also marked by significant CaM expression (Fig 2E, arrows). The same was true for the cerebellum. In particular, Purkinje cell dendrites relying on Cobl for branching [15] showed marked anti-CaM immunosignals (Fig 2F, arrows).

These findings suggested Cobl to be a Ca^2+^/CaM-regulated cytoskeletal effector. Indeed, the Cobl/CaM interaction was also observable in coprecipitation analyses with endogenous Cobl from rat brain lysates. The Cobl/CaM interaction was strongly Ca^2+^dependent and occurred in a wide range of Ca^2+^ concentrations (tested were physiological concentrations down to 2 μM) (Fig 2G).

We next aimed at further corroborating the Cobl/CaM interaction by coimmunoprecipitations of the endogenous proteins with suitable anti-CaM antibodies. A subpool of endogenous Cobl indeed was specifically coimmunoprecipitated with CaM from rat brain lysate (Fig 2H).

### Cobl-Mediated Dendritic Arborization Requires Ca^2+^/CaM Signals

**Fig 3:**
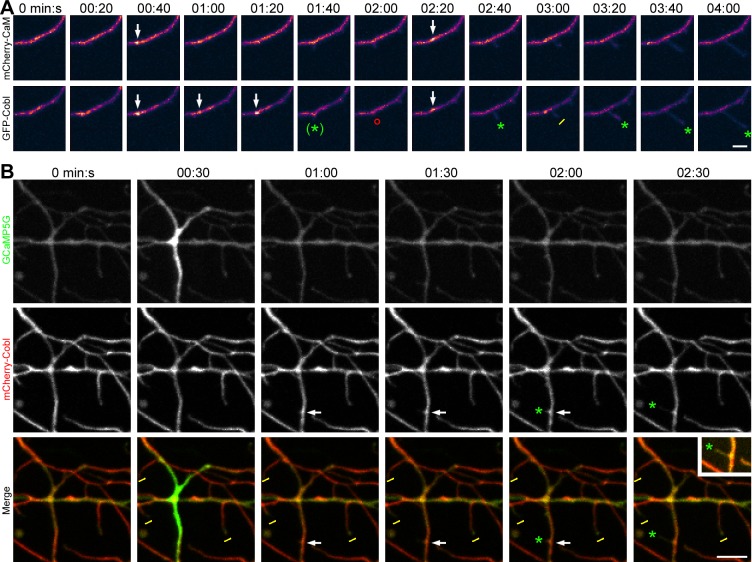
Correlation of Cobl and CaM dynamics and Ca^2+^ signals at forming dendritic protrusions. (**A**) MIPs from 3-D-time-lapse recordings of GFP-Cobl and mCherry-CaM in a dendrite of a primary hippocampal neuron transfected at DIV6 and imaged at DIV7 show that episodes of Cobl accumulation at distinct dendritic sites as well as the induction of protrusions from such sites are accompanied by accumulations of CaM at the same sites (data shown as heat map of fluorescence intensities from purple to white, see Fig 1 for legend of color coding; see S4 Fig for original data of both channels and for merged images). Bar, 2 μm. Please also see S3 Movie. (**B**) MIPs from 3-D-time-lapse recordings of the calcium sensor GCaMP5G and mCherry-Cobl reveal that local rises in calcium levels coincide with subsequent induction of Cobl accumulation and dendritic protrusions arising from Cobl-enriched sites (arrows). Bar, 5 μm. Inset is a high-intensity image of the protrusion. Cobl and CaM accumulations, respectively, at sites giving rise to protrusion are marked by arrows. Green *, protrusion that was initiated or grew when compared to previous image; (green *), minor, aborted protrusion; red **°**, protrusion that shrunk compared to previous image; yellow I, static protrusion.

We next examined whether Cobl-enriched sites in dendrites may represent sites of Ca^2+^/CaM signaling. 3-D-time-lapse studies showed a dynamic coenrichment of Cobl and CaM at sites of protrusion initiation (Fig 3A; S4 Fig; S3 Movie).

Calcium imaging using GCaMP5G showed that also Ca^2+^ signals correlated with both Cobl accumulation and protrusion initiation (Fig 3B, arrow).

**Fig 4:**
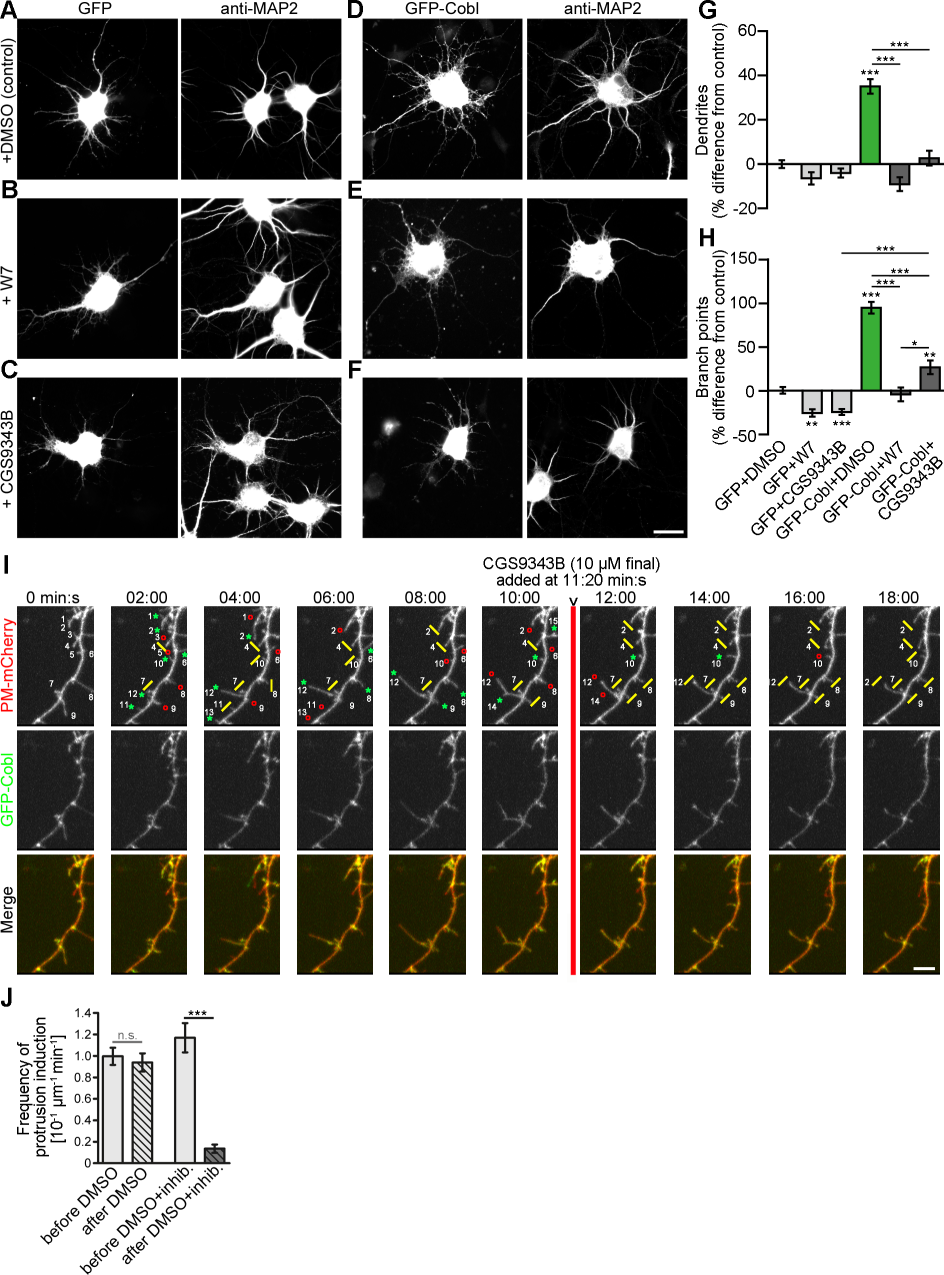
Cobl-mediated dendritogenesis is suppressed by CaM inhibition. (**A–H**) End-point analysis of CaM inhibitor effects in primary hippocampal neurons (DIV4+2). (**A–F**) Hippocampal neurons transfected as indicated at DIV4 and treated with 0.1% (final) DMSO (**A,D**), CaM inhibitor W7 (**B,E**) and CaM inhibitor CGS9343B (both in 0.1% DMSO final) (**C,F**), respectively, 30 h after transfection. Cells were processed for anti-MAP2 immunostaining 2 d after transfection. Bar, 20 μm. (**G,H**) Quantitative examinations of dendrite number (**G**) and dendritic branch points (**H**) normalized to the GFP+DMSO controls of each assay. Note that Cobl-mediated dendrite formation is suppressed by the CaM inhibitors W7 and CGS9343B. Data represent mean ± SEM. GFP+DMSO, *n* = 197; GFP+W7, *n* = 120; GFP+CGS9343B, *n* = 169; GFP-Cobl+DMSO, *n* = 101; GFP-Cobl+W7, *n* = 53; GFP-Cobl+CGS93943B, *n* = 80. Statistical significances (to control, marked above column; between other conditions, indicated by lines) were determined using one-way ANOVA with Tukey’s post-test. *p < 0.05; **p < 0.01; ***p < 0.001. (**I**) 3-D-time-lapse imaging of GFP-Cobl dynamics and neuronal morphogenesis visualized by PM-mCherry before and after incubation with the CaM inhibitor CGS9343B. Shown is a selection (times as indicated) of MIPs recorded by spinning disc microscopy. Green *, protrusion that was initiated or grew when compared to previous image (examples numbered to allow for tracking); red **°**, protrusion that shrunk compared to previous image; yellow I, static protrusion. Note that whereas most neuronal structures were dynamic, CGS9343B addition (red line; 11:20 min:s) impeded this morphological dynamics and neuronal structures largely were static until the end of recording. Bar, 5 μm. Please also see S4 Movie. (**J**) Quantitation of the frequencies of protrusion initiation before and after addition of DMSO and DMSO+inhibitor (CGS9343B), respectively. DMSO, *n* = 12; DMSO+inhibitor, *n* = 10 dendrite sections. For data underlying **G, H,** and **J** see S1 Data. Statistical significances, one-way ANOVA with Tukey’s post-test. ***p < 0.001.

To experimentally test the effects of Ca^2+^/CaM signals on Cobl’s functions in dendritogenesis, we next employed two different CaM inhibitors: W7 (N-(6-Aminohexyl)-5-chlor-1-naphthalinsulfonamide) [17] and CGS9343B (1,3-dihydro-l-[1-[4-methy1-4H,6H-pyrrolo[1,2-a][4,l]-benzoxazepin-4-y1-methy1]-4-piperidinyl]-2H-benzimidazol-2-one(1:1) maleate) [18] (Fig 4). W7 and CGS9343B both completely suppressed Cobl-induced dendritic arborization in developing neurons (Fig 4A–4H). Also in control cells, W7 and CGS9343B incubation caused some reduction of dendritic branch points. These effects were smaller than the strong suppression of the Cobl overexpression phenotype indicating that the CaM inhibitors indeed suppress Cobl functions and do not act in a parallel pathway. The effect of W7 and CGS9343B in control cells may include an inhibition of the functions of endogenous Cobl that has been demonstrated to be a crucial factor in dendritic arborization [12] and is expressed throughout the development of the brain (S5 Fig). Similar to dissociated neurons, significant reductions of dendritic branch points by W7 and CGS9343B were also observed in Purkinje cells in developing cerebellar slice cultures (S6 Fig). Interestingly, the observed impairments mirror quite well the Coblloss-of-function phenotype in cerebellar slices [15].

Highlighting the neuronal morphology of primary rat hippocampal neurons at days in vitro (DIV)6 with PM-mCherry in 3-D-time-lapse studies showed many dynamic protrusions originating from Cobl-enriched sites in untreated neurons. After addition of CGS9343B, this dynamic behavior rapidly came to a standstill. Neuronal structures became static, and Cobl-enriched sites appeared less frequently (Fig 4I; S4 Movie). Quantitative analyses showed that the frequencies of protrusion initiation decreased to about 10% of the values of the respective cells before addition of CaM inhibitor (Fig 4J).

Taken together, Cobl-mediated dendrite branching requires Ca^2+^/CaM signaling events.

### Ca^2+^ Promotes the G-actin Binding of Cobl, While Ca^2+^/CaM Attenuates the Actin Binding of the First WH2 Domain

**Fig 5:**
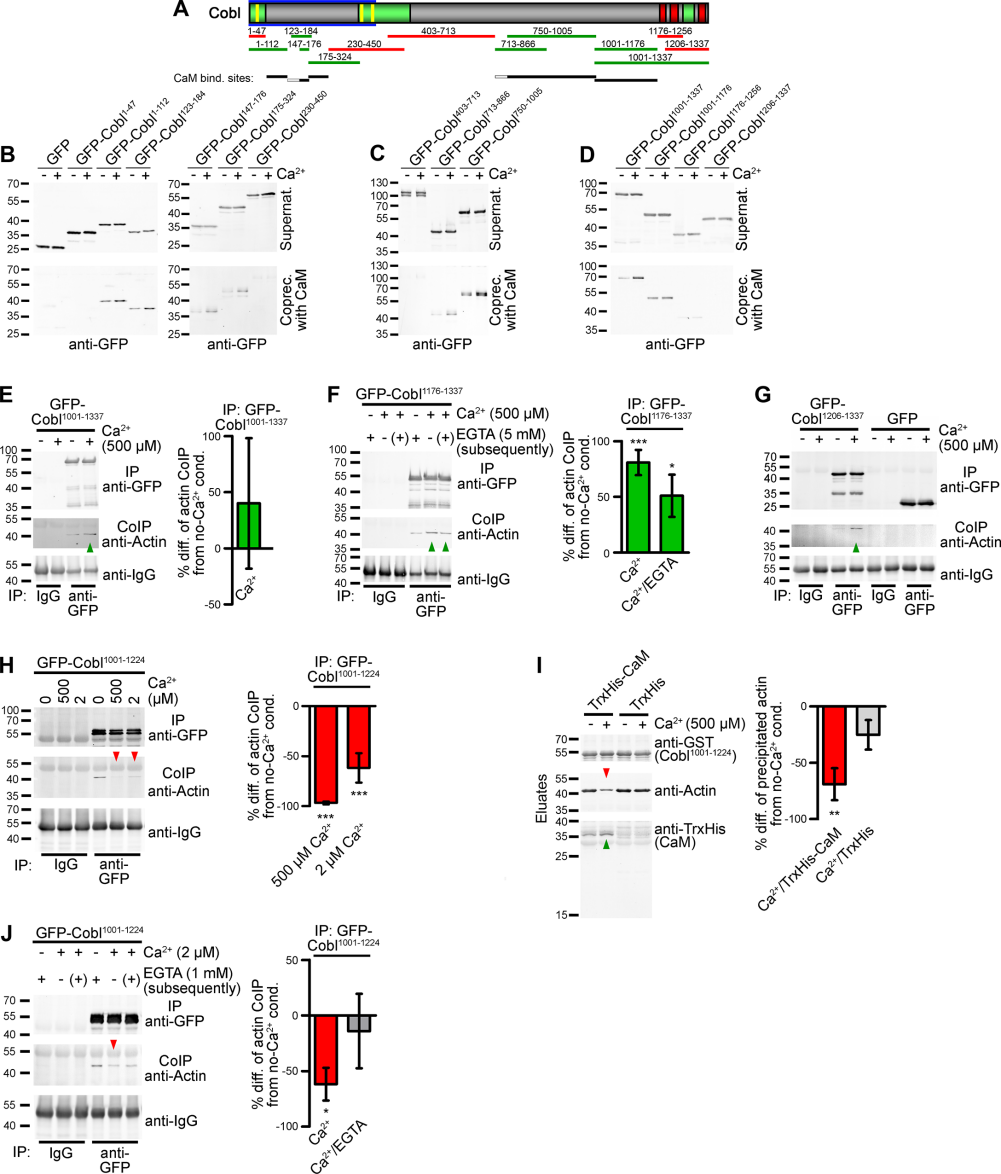
Cobl has multiple CaM binding sites and Ca^2+^/CaM controls the actin binding activity of Cobl’s WH2 domains. (**A**) Scheme of Cobl showing proline-rich domains (green), WH2 domains (red), Cobl Homology domain (blue frame), and syndapin-binding KrRAPpPP-motifs (yellow). Deletion mutants binding to CaM are in green, those not binding in red. Black lines, (independent) CaM-binding interfaces (grey lines, binding not addressed individually). (**B–D**) Coprecipitation experiments with immobilized CaM and GFP-Cobl deletion mutants in presence and absence of 500 μM Ca^2+^-identifying CaM binding sites within the N-terminus (**B**), the C-terminal half (**C**) and the WH2 domain-containing C-terminus (**D**). (**E–G**) Immunoprecipitations of GFP-Cobl^1001–1337^, GFP-Cobl^1176–1337^ and GFP-Cobl^1206–1337^ all show an increase of coimmunoprecipitation of endogenous actin upon Ca^2+^ addition. Quantitative data (right panels in **E** and **F**) are mean percental differences±SEM from the respective Ca^2+^-free conditions. GFP-Cobl^1001–1337^, *n* = 4; GFP-Cobl^1176–1337^, Ca^2+^, *n* = 5; Persistence upon subsequent addition of 5 mM EGTA (Ca^2+^/EGTA), *n* = 3. (**H**) Quantitative coimmunoprecipitations demonstrating that actin association by a combination of the first WH2 domain and the CaM interface is inhibited upon Ca^2+^ addition at both 500 μM (*n* = 6) and 2 μM Ca^2+^ (*n* = 4). (**I**) In vitro reconstitution of Cobl^1001–1224^/CaM/actin complexes with purified proteins (GST-Cobl^1001–1224^ immobilized) demonstrating the Ca^2+^ and TrxHis-CaM dependence of the observed suppression of the actin binding of the first WH2 domain of Cobl (for input of purified proteins see S11 Fig). Right panel, quantitative analyses, *n* = 3 each. (**J**) Reversibility of the actin binding to GFP-Cobl^1001–1224^ suppressed by Ca^2+^ via subsequent addition of EGTA (2 μM Ca^2+^/1 mM EGTA). 2 μM Ca^2+^, *n* = 4; 2 μM Ca^2+^/1 mM EGTA, *n* = 2. For data underlying **E, F,** and **H–J,** see S1 Data. Statistical significances in all panels, one-way ANOVA with Tukey’s post-test. *p < 0.05, **p < 0.01, ***p < 0.001.

To unravel the mechanisms of CaM’s crucial role in Cobl-mediated dendritogenesis, we next mapped the CaM binding sites. Surprisingly, we identified multiple CaM interactions with Cobl. The N-terminus contains at least three independent CaM-binding areas (aa48-112, 147–176 and 175–229) (Fig 5A and 5B).

Coprecipitation and corecruitment studies furthermore showed that CaM binding additionally involved regions in the middle of Cobl (aa750-1005) (Fig 5C) as well as in front of the C-terminal WH2 domains (aa1001-1176) (Fig 5D; S7 Fig). Half-maximal binding was reached at 0.68 and 0.95 μM Ca^2+^ for N- and C-terminal parts of Cobl, respectively (S8 Fig). These values are in line with the cooperative Ca^2+^ binding of CaM, the k_D_ of which decreases to ~0.5 μM upon target binding in the case of CaMKII (NM_009792.3; GI:161086915) [19–21].

Our observations raised the exciting possibility that Ca^2+^/CaM modulates Cobl’s cytoskeletal functions. As neither full-length Cobl nor extended C-terminal parts of Cobl can be purified [12,14], and in vitro reconstitutions of actin polymerization are hampered by Ca^2+^-containing physiological buffers, we focused on actin coimmunoprecipitations and in vitro reconstitutions of actin binding to explore putative Ca^2+^/CaM-induced modulations of Cobl functions.

Coimmunoprecipitation of endogenous actin with GFP-Cobl^1001–1337^, GFP-Cobl^1176–1337^, and GFP-Cobl^1206–1337^ unveiled that Ca^2+^ promotes actin binding. This positive effect was independent of CaM binding and also independent of the first WH2 domain neighboring the CaM binding interface but prominently involved the second and third WH2 domain of Cobl (Fig 5E–5G). Interestingly, the Ca^2+^-mediated increase of actin binding was not reversed upon subsequent lowering of Ca^2+^ levels (Fig 5F).

Since the increased actin association was more striking for GFP-Cobl^1176–1337^ lacking the CaM binding site than for GFP-Cobl^1001–1337^ containing an interface for direct CaM binding (S9 Fig), we next addressed the effects of Ca^2+^/CaM specifically on the first WH2 domain. Interestingly, the first WH2 domain (Cobl^1176–1224^) required the CaM-binding region (Cobl^1001–1176^) for coimmunoprecipitation of actin (S10 Fig). This specific coimmunoprecipitation of actin by Cobl^1001–1224^ was strongly suppressed by 500 μM Ca^2+^, which may only be reached in direct vicinity of Ca^2+^ channels, as well as by lower Ca^2+^ levels (2 μM), which are more commonly and more widely reached in neurons (Fig 5H).

Importantly, in vitro reconstitutions with purified proteins demonstrated that this suppression of actin binding of Cobl’s first WH2 domain solely involved actin, Cobl and Ca^2+^/CaM. In the presence of CaM, we observed a Ca^2+^-specific CaM/Cobl complex formation and a statistically significant reduction of actin binding. In contrast, in the absence of CaM, no such difference between Ca^2+^ and Ca^2+^-free conditions was observed (Fig 5I; S11 Fig). Thus, the suppression of the actin binding of the first WH2 domain involves direct Ca^2+^/CaM association and relies on the Ca^2+^ sensor CaM.

The first WH2 domain is crucial for Cobl’s actin filament promoting function [12,14], and actin filament formation as well as Cobl accumulation was observed at the initiation point of Ca^2+^-triggered, newly forming dendritic protrusions (Fig 1). Both observations strongly suggested that actin filament formation at branch initiation sites plays a supporting role during dendritic arborization. It was therefore interesting that Ca^2+^ was overall promoting actin association of the Cobl C-terminus, despite a simultaneously occurring Ca^2+^/CaM binding-mediated suppression of the actin association of the first WH2 domain. This suggested that actin binding may be transiently increased even further once Ca^2+^ levels drop again, as under such conditions the dissociation of CaM may release the suppression. In line with a transient effect, the Ca^2+^/CaM-mediated block of actin binding to the first WH2 domain indeed was fully reversible upon reduction of Ca^2+^ levels (Fig 5J).

### Ca^2+^/CaM Regulation of Cobl Homology Domain-Mediated Membrane Association

**Fig 6:**
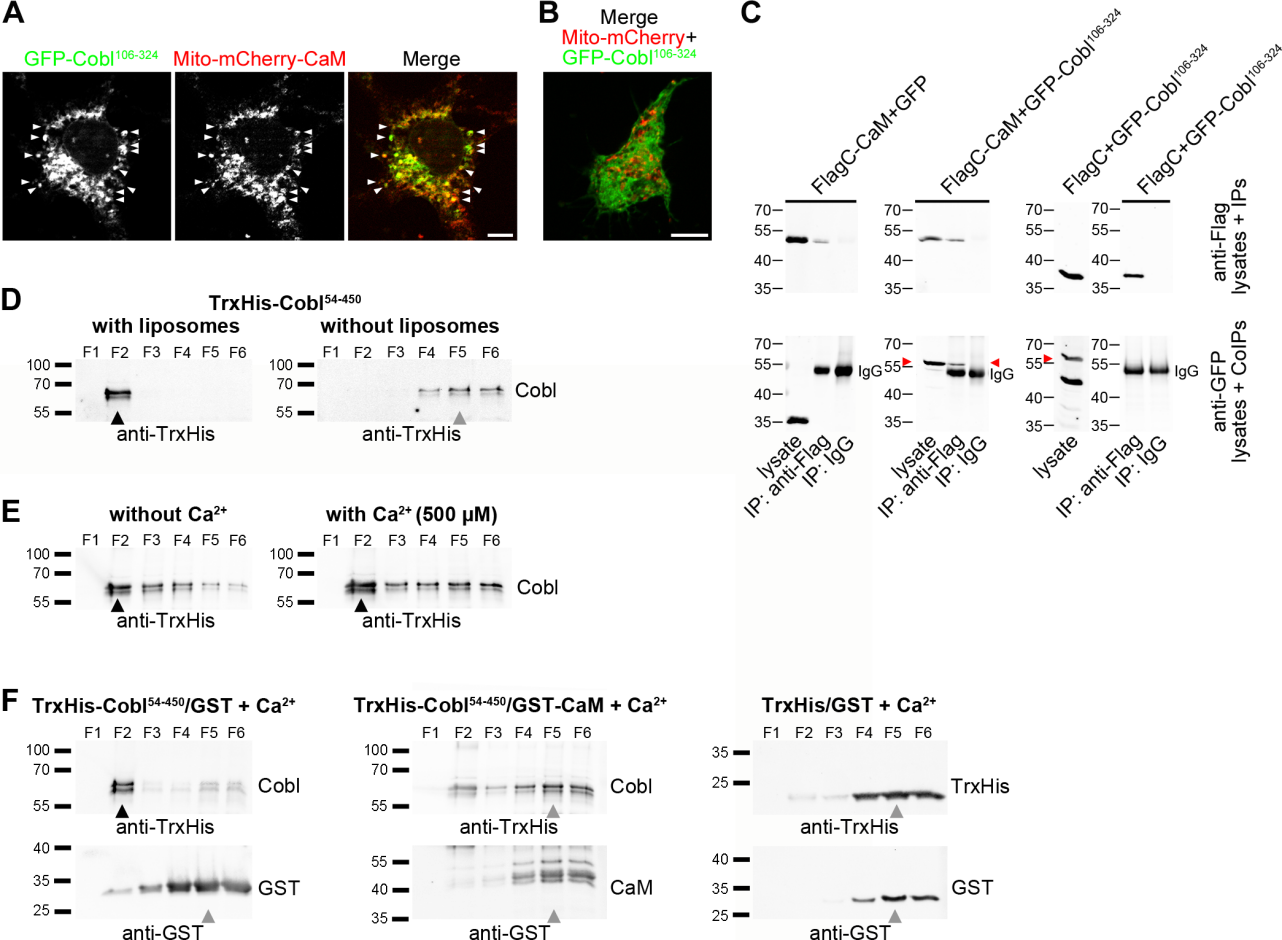
Ca^2+^/CaM associates with the Cobl Homology domain in vivo, and this association suppresses membrane binding of the Cobl Homology domain. (**A,B**) CaM complex formation with Cobl in intact cells demonstrated by GFP-Cobl^106–324^ recruitment to mitochondria coated with mCherry-CaM (Mito-mCherry-CaM) (**A**; arrowheads mark examples) but not to those with mCherry alone (**B**; Mito-mCherry control). Bars, 10 μm. (**C**) Coimmunoprecipitation experiments with Flag-mCherry-CaM (FlagC-CaM) and GFP-Cobl^106–324^ expressed in HEK293 cells further confirming the interaction in vivo. Red arrowheads mark GFP-Cobl^106–324^ coimmunoprecipitated with FlagC-CaM (see middle lane of middle panel). Bands of sizes smaller than 57kD in lysates (GFP-Cobl106-324) are degradation products. (**D**) Immunoblot analyses of density gradient fractions (F1, top; F6, bottom) from floatation assays with liposomes made of Folch fraction I. TrxHis-Cobl^54–450^ was incubated with and without liposomes. Note that TrxHis-Cobl^54–450^ has a specific membrane-binding activity and floats together with the liposomes to density gradient fraction 2. (**E**) TrxHis-Cobl^54–450^ floatation assays with and without calcium revealing that Cobl’s membrane-binding ability is Ca^2+^insensitive. (**F**) Floatation assays examining the ability of TrxHis-Cobl^54–450^ to bind to liposomes in the presence of CaM. TrxHis and GST were used to control for putative unspecific interactions. Note that presence of Ca^2+^/CaM strongly and specifically suppresses the lipid association of TrxHis-Cobl^54–450^, and that both proteins remain at the bottom of the gradients. Black arrow heads in **D–F** mark protein bands at floating liposome positions (density gradient fraction 2), and grey arrow heads mark protein bands that remain in bottom fractions, the maximum of which usually is in fraction 5.

The observation that CaM inhibition impaired Cobl accumulation at branch initiation sites (Fig 4I and 4J) prompted us to study the influence of Ca^2+^/CaM on Cobl’s association with the cell cortex. This process involves Cobl Homology domain interactions with syndapin I [16]. Specific coimmunoprecipitation as well as specific reconstitutions of Cobl–CaM interactions in intact cells confirmed that also the CaM interactions with the Cobl Homology domain are of relevance in vivo (Fig 6A–6C).

We thus addressed the exciting hypothesis that Ca^2+^/CaM signaling may not only control Cobl’s actin cytoskeletal functions but may also orchestrate Cobl’s membrane association. Whereas we were unable to purify the full Cobl Homology domain, we succeeded in purifying an alternative protein (TrxHis-Cobl^54–450^). In vitro reconstitutions with liposomes revealed that the N-terminus of Cobl itself has membrane-binding activity and therefore floated with liposomes irrespective of calcium presence (Fig 6D and 6E).

Interestingly, Ca^2+^/CaM addition effectively suppressed the direct membrane-binding ability of the Cobl Homology domain (Fig 6F; upper middle panel).

### Ca^2+^/CaM Promotes Cobl/Syndapin I Complex Formation

**Fig 7:**
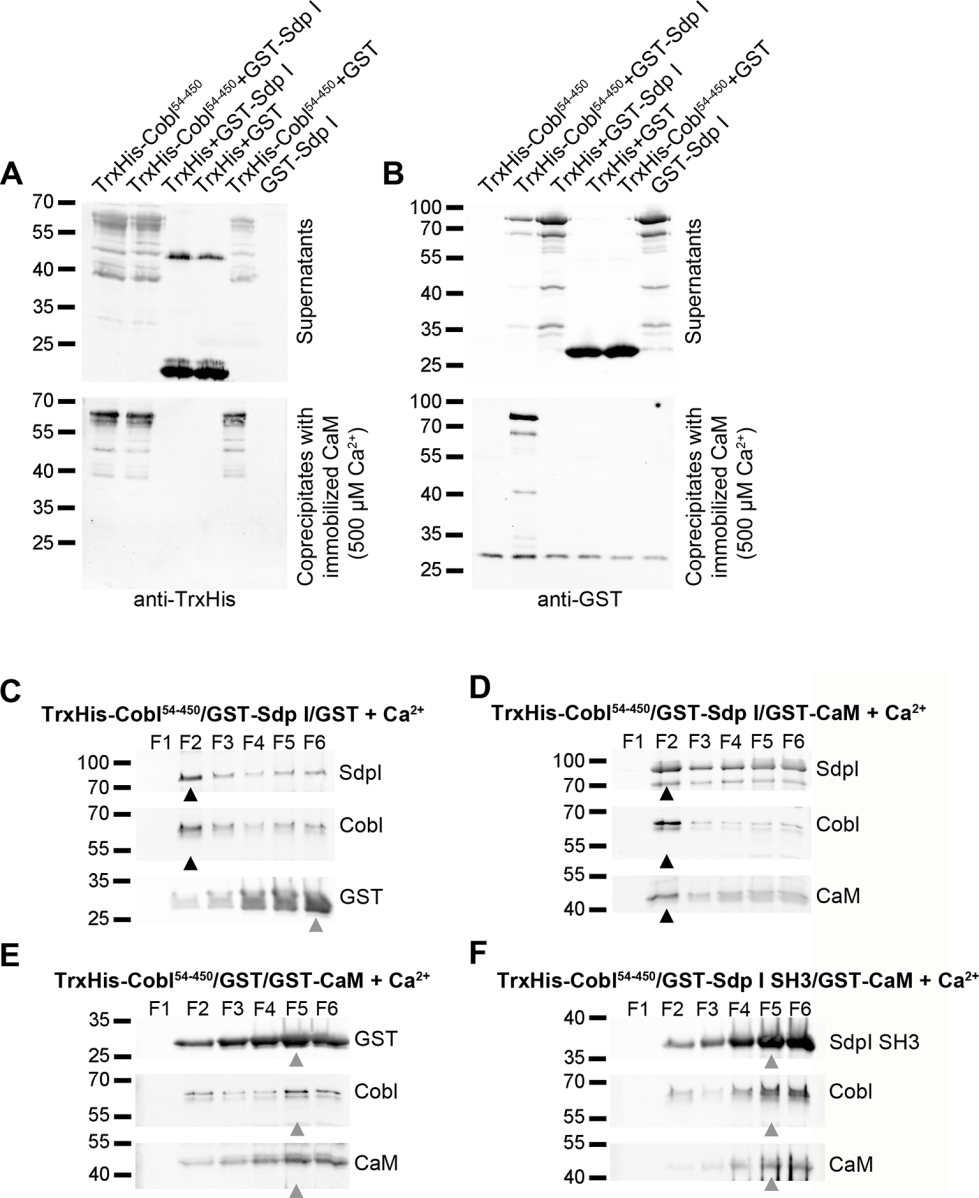
Complexes composed of the three components syndapin I, Cobl, and CaM form and associate with liposomes. (**A,B**) Immunoblot analyses of coprecipitation experiments using purified, immobilized CaM, purified TrxHis-Cobl^54–450^ and GST-syndapin I demonstrate that Cobl/CaM complexes but not CaM alone specifically interact with syndapin I. (**A**) Direct interaction of Cobl^54–450^ with immobilized CaM. (**B**) Syndapin I is present in Cobl/CaM complexes in a Cobl-dependent manner (i.e., does not associate with CaM). Thus, Cobl can directly and simultaneously associate with both CaM and syndapin I. (**C–F**) Liposome floatation assays evaluating the lipid association of Cobl, CaM, and syndapin I when combined. Note that TrxHis-Cobl^54–450^ and GST-syndapin I float in the presence of both GST (**C**) and GST-CaM (**D**) and that in the latter case, CaM also floats together with Cobl and syndapin I to fraction 2. In contrast, in both Cobl/CaM combinations that either lack syndapin I (**E**; TrxHis-Cobl^54–450^/GST/GST-CaM) or instead of syndapin I full-length only contain the Cobl-binding SH3 domain of syndapin I, i.e., lacking the ability of syndapin I to directly bind to lipids (**F**), neither Cobl nor CaM floated (**E,F**). Grey arrow heads (**C,E,F**) mark protein bands that remain in bottom fractions, the maximum of which usually is in fraction 5, and black arrow heads (**C,D**) mark protein bands at floating liposome positions (density gradient fraction 2).

Cortical targeting of cytoskeletal effectors is a key aspect in shaping cells. The Ca^2+^/CaM-mediated suppression of Cobl’s direct lipid association thus was puzzling. We therefore analyzed a putative regulation of Cobl/syndapin I interactions. In vitro reconstitutions demonstrated that direct and simultaneously occurring interactions of Cobl with CaM and syndapin I give rise to complexes containing all three components (Fig 7A and 7B).

Strikingly, the anchoring of Cobl to membranes via the F-BAR domain protein syndapin I was not suppressed by Ca^2+^/CaM addition. Complexes containing all three components, i.e., Cobl, CaM, and syndapin I, floated with liposomes (Fig 7C–7E).

Together, the formation of complexes composed of all three components and their ability to associate with membranes suggested that Cobl’s intrinsic lipid association constitutively ensures some Cobl presence at the cell cortex. Upon association of Ca^2+^/CaM, this ability of Cobl is switched off. As a consequence, F-BAR domain–mediated membrane associations by syndapin I [22,23] start to dominate the spatial control of Cobl at the cell cortex. Consistent with such a scenario, we observed that addition of the Cobl-binding SH3 domain of syndapin I did not suffice for restoring Cobl’s membrane association in the presence of Ca^2+^/CaM (Fig 7F).

Thus, upon Ca^2+^/CaM association, Cobl localization becomes fully dependent on SH3 domain interactions and on F-BAR-mediated membrane association of syndapin I.

**Fig 8:**
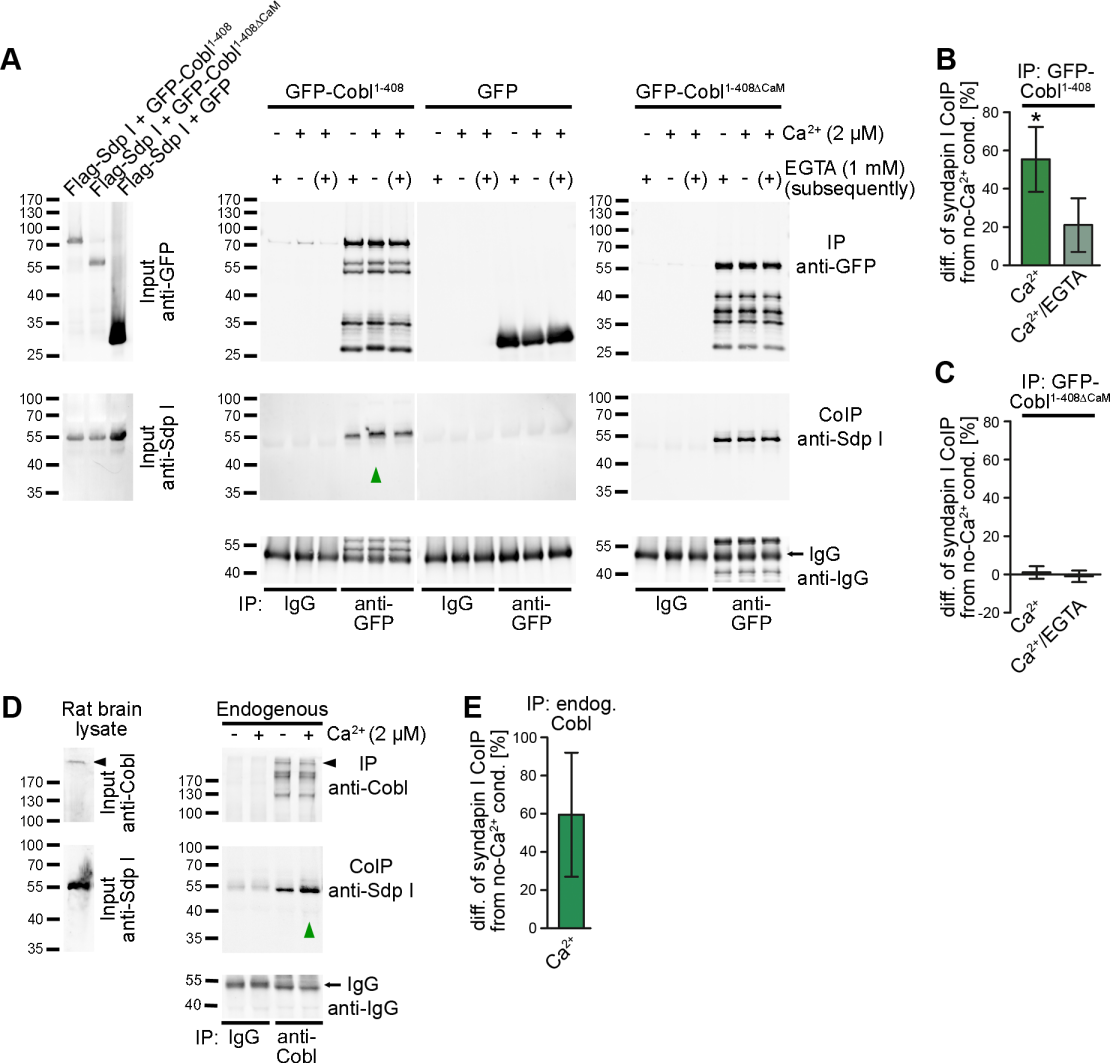
Ca^2+^/CaM association with the Cobl Homology domain of Cobl promotes Cobl/syndapin I complex formation. (**A**) Coimmunoprecipitations of GFP-Cobl^1-408^/Flag-syndapin I (Flag-Sdp I) and GFP-Cobl^1-408∆CaM^/Flag-syndapin I complexes from HEK293 cells under Ca^2+^-free conditions (−), 2 μM Ca^2+^ (+) and 2 μM Ca^2+^ with subsequent incubation with EGTA (2 μM Ca^2+^/1 mM EGTA, [+]). The syndapin I interaction with Cobl is promoted upon activation of Ca^2+^ signaling (2 μM Ca^2+^) (indicated by the green upright arrowhead). For further confirmation of the Ca^2+^/CaM association-dependent increase of the syndapin I interaction, also see coimmunoprecipitations under alternative conditions shown in S12 Fig. (**B,C**) Quantitative evaluations of coimmunoprecipitated syndapin I normalized to immunoprecipitated Cobl and displayed as mean percental difference ± SEM from the respective Ca^2+^-free conditions show that the syndapin I interaction is reversibly promoted by increasing calcium. Note that syndapin I coimmunoprecipitation by the CaM binding-deficient Cobl mutant Cobl^1-408∆CaM^ is insensitive to changes of calcium levels. The observed regulation of Cobl/syndapin I complex formation thus requires the CaM binding interface of Cobl. GFP-Cobl^1-408^/Flag-Sdp I +/− Ca^2+^, *n* = 3 each; with Ca^2+^/EGTA, *n* = 2. GFP-Cobl^1-408∆CaM^/Flag-Sdp I +/− Ca^2+^, *n* = 3 each; with Ca^2+^/EGTA, *n* = 2. Statistical significances were calculated by one-way ANOVA with Tukey’s post-test. *p < 0.05. (**D,E**) Coimmunoprecipitations from rat brain lysates (**D**) demonstrate the promotion of endogenous Cobl/syndapin I complexes upon calcium addition (highlighted by green upward arrowhead; **E,** quantitation; *n* = 2. For data underlying **B, C,** and **E,** see S1 Data.

This regulatory mechanism would be even more effective if syndapin I associations were promoted upon Ca^2+^/CaM. In order to address this directly in vivo, we conducted quantitative coimmunoprecipitation studies. We observed a Ca^2+^-mediated increase of Cobl^1-408^/syndapin I complex formation at both 500 μM (not shown) and 2 μM calcium (+55.3 ± 17.0%; *p* < 0.05) when compared to conditions without calcium (Fig 8A and 8B; S12 Fig).

This increase was, to a large extent, reversible. At least upon prolonged incubation with ethylenglycol-bis(aminoethylether)-N,N,N′,N′-tetraacetic acid (EGTA) after Ca^2+^ stimulation, syndapin I coimmunoprecipitation intensities only remained moderately increased and were not significantly different from control anymore (+21.0 ± 11.4%; Fig 8A and 8B).

The striking promotion of Cobl’s association with syndapin I was dependent on Cobl’s ability to associate with the calcium sensor CaM because a Cobl mutant incapable of binding to CaM (Cobl^1-408∆CaM^) did not respond to changes of Ca^2+^ levels. Instead, Cobl^1-408∆CaM^ coimmunoprecipitated constant amounts of syndapin I (Fig 8A and 8C).

Endogenous coimmunoprecipitations from rat brain lysates showed a corresponding calcium-mediated increase of Cobl/syndapin I complex formation that was consistent with the ~60% increase of syndapin I association in the heterologous coimmunoprecipitations using the Cobl Homology domain described before (Fig 8D and 8E).

### Syndapin I Coincides with Cobl Accumulations at Sites of Dendritic Membrane Protrusion

**Fig 9:**
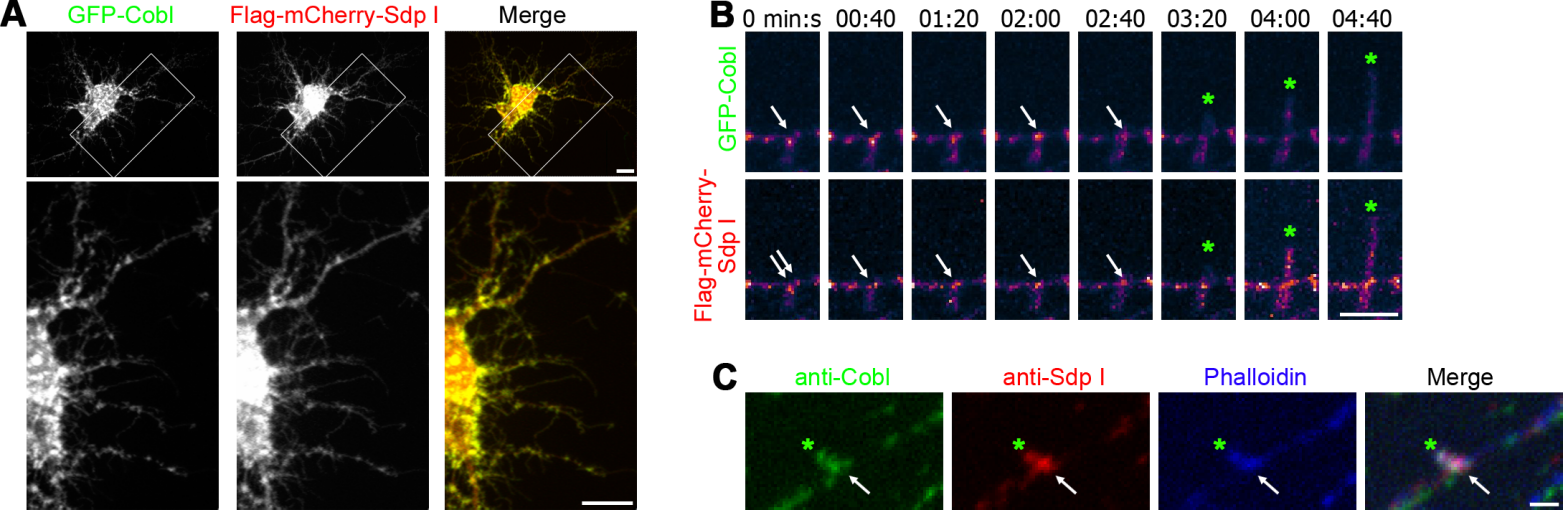
Syndapin I and Cobl localize to sites of dendritic branch initiation. (**A**) Colocalization of GFP-Cobl and Flag-mCherry-Sdp I at discrete sites within the dendritic arbor of a DIV7 hippocampal neuron. Lower panels represent magnifications of boxed areas. Bars, 10 μm. (**B**) Time-lapse spinning disc MIPs of a dendritic protrusion (green *) emanating from a Cobl and syndapin I-enriched site (arrows). Data is shown as heat map of fluorescence intensities from purple to white, see Fig 1 for legend of color coding; for original fluorescence data and colocalization analyses, see S13 Fig. Bar, 5 μm. (**C**) Localization of endogenous Cobl at dendritic sites marked by accumulations of anti-syndapin I immunosignals and of F-actin (detected by phalloidin). Bar, 1 μm.

In primary neurons undergoing dendritogenesis (DIV7), GFP-Cobl and Flag-mCherry-syndapin I colocalized at discrete sites within the dendritic arbor (Fig 9A). 3-D-time-lapse recordings showed that protrusions emanated from sites that were enriched for both syndapin I and Cobl. Both proteins showed very good spatial overlap at nascent dendritic branch points. Similar to the dynamic behavior of Cobl during dendritic branch induction, also syndapin I was found to accumulate at sites of branch initiation shortly before branch induction started. After protrusions had been established and grew, syndapin I and Cobl both redistributed to a more disperse localization in the mother dendrite and in the formed branch (Fig 9B; S13 Fig).

Immunostainings of endogenous Cobl and syndapin I in developing neurons confirmed the presence of syndapin I-enriched sites in dendrites that also showed accumulations of anti-Cobl signals. Often such sites were not symmetric but protruded from one side of the dendrite and may thus represent initiation sites for dendritic branching (Fig 9C).

### CaM Association Is Critical for Cobl-Mediated Dendrite and Dendritic Branch Formation

**Fig 10:**
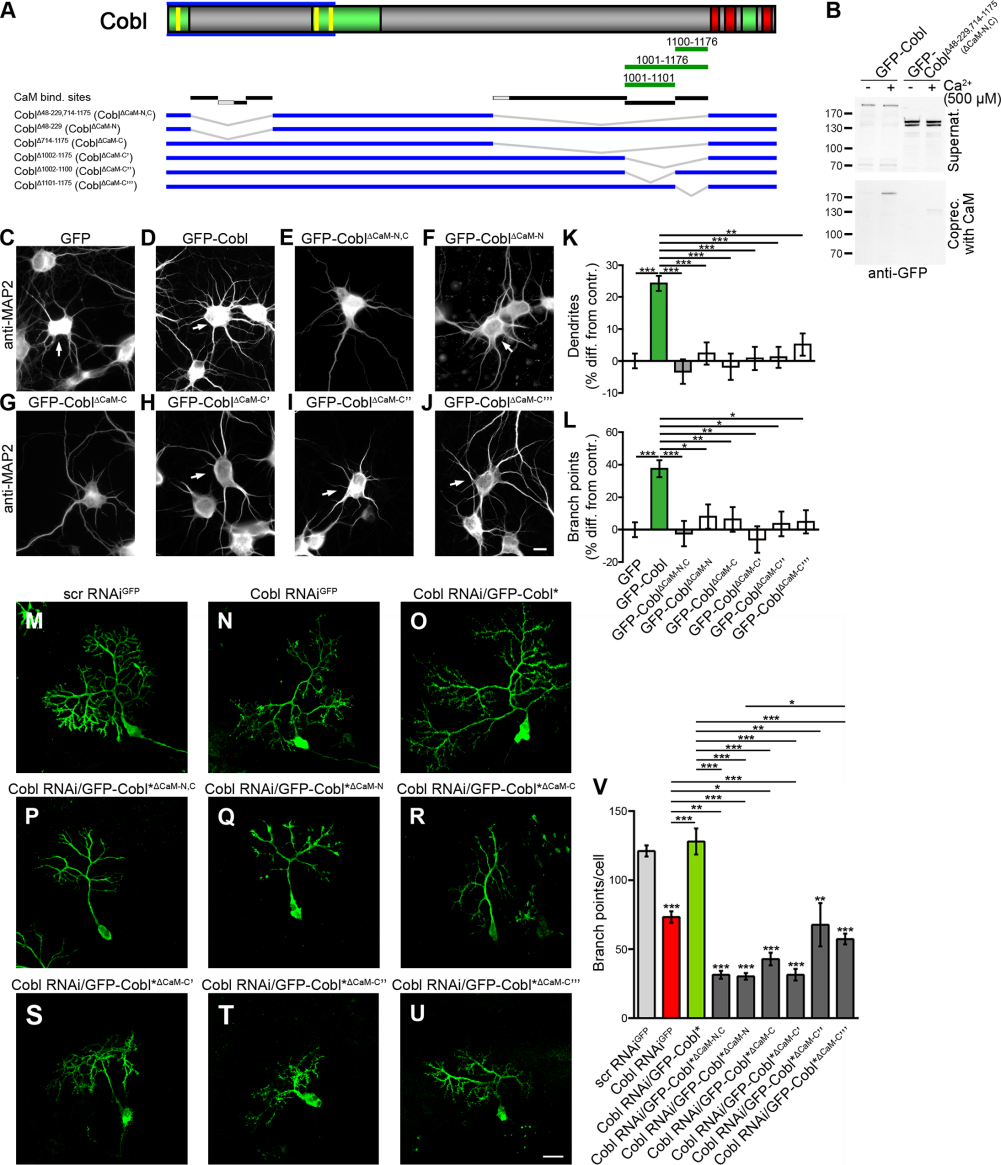
Cobl functions in dendritogenesis rely on CaM association with both the Cobl N- and C-terminus. (**A**) Scheme of Cobl as in Fig 5 with a schematic representation of further pieces of Cobl positively tested for CaM association (green lines; for immunoblottings of coprecipitation analyses see S14 Fig), an overview of the identified CaM binding interfaces (black lines; grey lines, binding not addressed individually) and Cobl mutants lacking CaM interfaces (blue lines with grey deletions). (**B**) Coprecipitation experiments with immobilized CaM and GFP-Cobl deletion mutant GFP- Cobl^∆48–299,714–1175^ (Cobl^∆CaM-N,C^) in comparison to wild-type GFP-Cobl showing that Cobl^∆CaM-N,C^ lacking all identified CaM binding areas did not associate with immobilized Ca^2+^-activated CaM but remained in the supernatant. (**C–L**) Functional analyses of Cobl^∆CaM-N,C^, Cobl^∆CaM-N^, Cobl^∆CaM-C^, Cobl^∆CaM-C^’, Cobl^∆CaM-C^”, and Cobl^∆CaM-C^”‘ mutants for their ability to elicit Cobl-mediated dendrite and dendritic branch formation upon overexpression in primary hippocampal neurons transfected at DIV4. Representative images of neurons transfected as indicated, fixed 2 d after transfection and immunostained for MAP2. In images displaying several neurons, transfected cells are marked by arrows. Bar, 10 μm. Quantitative analyses normalized to GFP controls show that both dendrite (**K**) and dendritic branch formation (**L**) require both the N-terminal and the C-terminal CaM interaction areas. Data are mean ± SEM. GFP, *n* = 206; GFP-Cobl, *n* = 187; Cobl^∆CaM-N,C^, *n* = 91, GFP-Cobl^∆CaM-N^, *n* = 98; Cobl^∆CaM-C^, *n* = 117; Cobl^∆CaM-C^’, *n* = 52, Cobl^∆CaM-C^”, *n* = 63, Cobl^∆CaM-C”‘^, *n* = 64. Statistical significances were tested using one-way ANOVA with Tukey’s post-test. *p < 0.05, **p < 0.01, ***p < 0.001. (**M–U**) Parasagittal cerebellar slices (250 μm; DIV2) prepared from postnatal day 10 (P10) mice showing individual Purkinje cells transfected as indicated. Bar, 20 μm. (**V**) Quantification of dendritic branch points of Purkinje cells in cerebellar slice cultures showed that coexpression of Cobl RNAi/GFP-Cobl*^∆CaM-N,C^, Cobl RNAi/GFP-Cobl*^∆CaM-N^, Cobl RNAi/GFP-Cobl*^∆CaM-C^, Cobl RNAi/GFP-Cobl*^∆CaM-C^’, Cobl RNAi/GFP-Cobl*^∆CaM-C^”, and Cobl RNAi/GFP-Cobl*^∆CaM-C^”‘ failed to rescue the reduction of branch points observed upon Cobl RNAi, whereas re-expressing full-length Cobl (GFP-Cobl*) did rescue the Cobl loss-of-function phenotype (Cobl*, RNAi-resistant Cobl (amino acid sequence unchanged)). Data are mean ± SEM. Scrambled RNAi^GFP^, *n* = 50; Cobl RNAi^GFP^, *n* = 21; Cobl RNAi/GFP-Cobl*, *n* = 16; Cobl RNAi/GFP-Cobl*^∆CaM-N,C^, *n* = 7; Cobl RNAi/GFP-Cobl*^∆CaM-N^, *n* = 18; Cobl RNAi/GFP-Cobl*^∆CaM-C^, *n* = 10; Cobl RNAi/GFP-Cobl*^∆CaM-C^’, *n* = 11; Cobl RNAi/GFP-Cobl*^∆CaM-C^”, *n* = 3 and Cobl RNAi/GFP-Cobl*^∆CaM-C^”‘, *n* = 16 cells. For data underlying **K, L,** and **V,** see S1 Data. Statistical significances were tested using one-way ANOVA with Tukey’s post-test (comparisons to scrambled RNAi controls are shown above columns; all others as marked by lines). *p < 0.05, **p < 0.01, ***p < 0.001.

To address whether CaM association is crucial for orchestrating Cobl functions during dendritogenesis, we next decided to employ GFP-Cobl mutants lacking N- and C-terminal CaM-binding interfaces or combinations thereof (Fig 10A). The importance of the most C-terminal CaM-binding area identified (Fig 5) hereby was addressed in form of two separate mutants, as further biochemical experiments revealed that the interface Cobl^1001-1176^ contained at least two areas that independently interact with CaM (Cobl^1001-1101^ and Cobl^1100-1176^). This increased the number of independent CaM interface on Cobl to at least six (Fig 10A; S14 Fig).

Cobl^∆CaM-N,C^ lacked all CaM-binding areas that we had identified and consistently did not associate with CaM anymore (Fig 10B). The Cobl N-terminus lacking the CaM interfaces (Cobl^∆CaM-N^) still associated with the two other components that are known to bind to the Cobl Homology domain and are critical for the functions of Cobl in vivo, Abp1 and syndapin I [15,16] (S15 Fig). Thus, this mutant should allow for dissecting the known requirements of syndapin I and Abp1 association from a putative importance of CaM associations in functional studies. Importantly, despite preserved Abp1 and syndapin I interactions, Cobl^∆CaM-N,C^ overexpression did not result in the extensive dendritic arborization observed upon overexpression of wild-type Cobl. Thus, the N-terminal and the more C-terminal CaM-binding interfaces are crucial for inducing Cobl overexpression phenotypes (Fig 10C–10L).

We next tested the functional importance of the identified CaM interfaces individually. Similar to Cobl^∆CaM-N,C^, also the individual deletions Cobl^∆CaM-N^ and Cobl^∆CaM-C^ were unable to trigger dendritic arborization (Fig 10F, 10G, 10K and 10L). Thus, Cobl functions seem to require both CaM binding sites of the N-terminal part of Cobl, which we showed to be involved in modulating the syndapin I interactions (Fig 8), as well as C-terminal CaM binding sites, which we unraveled to modulate actin associations (Fig 5).

Cobl with even smaller deletions (Cobl^∆CaM-C^’, Cobl^∆CaM-C^”, and Cobl^∆CaM-C^”‘) also failed to give rise to the Cobl gain-of-function phenotype. Instead, the morphology of transfected neurons remained indistinguishable from control cells (Fig 10H–10L). Therefore, the CaM binding area Cobl^1001-1176^ addressed in our mechanistic studies of Ca^2+^/CaM-mediated actin association was critically required for Cobl-mediated dendritic branching.

To address whether these data reflect a requirement of both N- and C-terminal CaM binding areas for physiological Cobl functions, we next conducted Cobl loss-of-function experiments and corresponding rescue experiments (Fig 10M–10V). We subjected cerebellar slices to gene gun transfections with GFP-reported plasmids. As described previously [15], Cobl RNAi-impaired dendritic branching of Purkinje cells. This Cobl loss-of-function phenotype was rescued by resupplying the cells with RNAi-insensitive, wild-type Cobl (GFP-Cobl*) (Fig 10M–10O and 10V).

In contrast, substitution of Cobl with any of the four CaM-binding–deficient mutants Cobl^∆CaM-N,C^ Cobl^∆CaM-N^, Cobl^∆CaM-C^, and Cobl^∆CaM-C^’ did not only fail to rescue the Cobl loss-of-function phenotype, but impaired dendritogenesis further (Fig 10P–10S and 10V). Cobl^∆CaM-C^” and Cobl^∆CaM-C^”‘ also were unable to rescue the Cobl loss-of-function defects in dendritic branching in the developing slice cultures (Fig 10T–10V).

Thus, Cobl’s crucial role in dendritic branch induction critically relies on both the N- and the C-terminal CaM association sites, for which we have revealed the molecular mechanisms of Cobl regulation.

## Discussion

**Fig 11:**
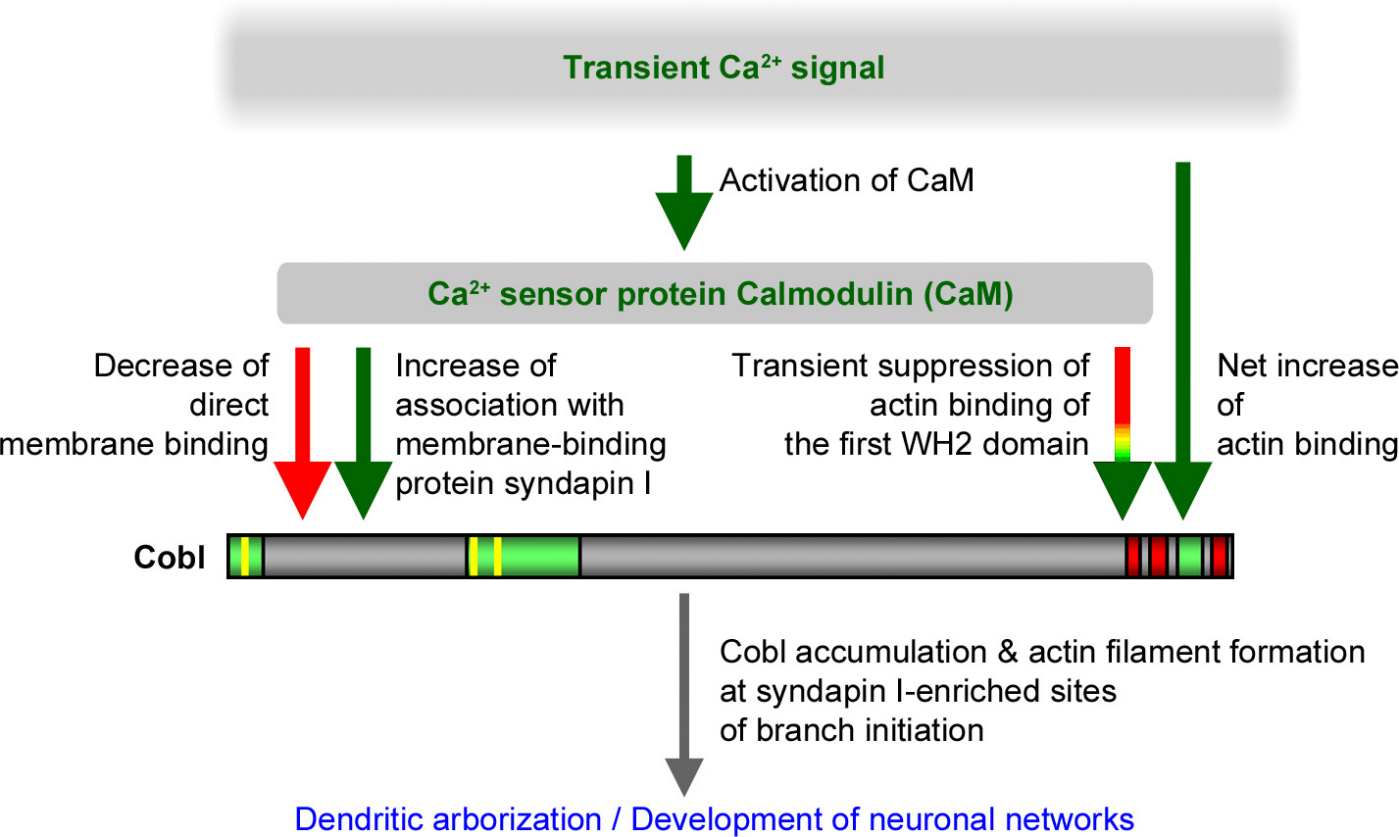
The signaling pathway and mechanisms revealed to control Cobl functions. Transient Ca^2+^ signals and CaM as a Ca^2+^ sensor protein control the functions of Cobl in neurons. Newly identified components of the pathway of Cobl-mediated actin filament formation in early neuronal development are in green, the molecular mechanisms experimentally demonstrated to be critical for the role of Cobl in branch induction are written in black. Colors of arrows indicate whether the mechanism identified to be important for Cobl-mediated function represents an inhibition or a promotion of the respective molecular property of Cobl.

Shaping neurons demands that Ca^2+^ signals are converted into locally restricted and transient activity of force-generating effectors [24]. The activity of such effectors must be targeted to the dendritic plasma membrane and must cease to exist once a branch is induced successfully. Whereas other actin cytoskeletal effectors are controlled by Rho-type GTPases, we here describe a direct interaction of the calcium sensor protein CaM with a powerful cytoskeletal effector remodeling neuronal morphology, the actin nucleator Cobl (Fig 11).

To our knowledge, only few direct links of Ca^2+^/CaM to the actin cytoskeleton have been discovered in neurons thus far. However, none of them explains how Ca^2+^ signals may trigger dendritogenesis. Neither the described CaM associations with brain-enriched spectrin isoforms [25] nor competitive binding of the F-actin bundling protein α-actinin and CaM to NMDA receptors and L-type Ca^2+^ channels [26,27] offer obvious mechanisms bringing about dendritic arborization.

With Cobl, we have identified a CaM-associating component that effectively promotes the local formation of actin filaments. Actin cytoskeletal forces have the power to shape cells and cellular compartments. Whether other WH2 domain-containing actin nucleators [10,11] or actin filament formation via the Arp2/3 complex and/or Formins also are directly controlled by Ca^2+^/CaM remains to be addressed.

We found by yeast-2-hybrid, coprecipitations, coimmunoprecipitations, and corecruitment studies in intact cells that CaM associates with Cobl. Reconstitutions with purified proteins demonstrated that Cobl’s interactions with CaM are direct. The Ca^2+^ dependency of the interactions strongly suggested that Cobl/CaM complexes are involved in translating Ca^2+^ signals sensed by CaM into cellular answers. Colocalizations in different parts of the brain and a dynamic coappearance of Cobl and CaM at induction sites of dendritic protrusion support this hypothesis.

Several lines of evidence from functional studies underscore the importance of Cobl/CaM interactions during dendritogenesis: CaM inhibitors fully suppressed Cobl-mediated dendrite formation and branching in quantitative end-point analyses of fixed neurons and in live imaging studies. Our data strongly argue that a direct association and not merely CaM signaling is required, as overexpression of Cobl mutants incapable of associating with CaM failed to mimic Cobl-mediated effects on dendrite formation. Furthermore, re-expression of such Cobl mutants failed to rescue the Cobl loss-of-function phenotype in dendritic branching of cerebellar Purkinje cells. These findings clearly point to a critical function of CaM associations with Cobl.

Our analyses revealed multiple independent CaM binding areas. At least three of them reside in the Cobl Homology domain. In addition, at least three further binding motifs are located in Cobl’s C-terminal part. Usually, the CaM lobes wrap around target segments, burying them in a hydrophobic channel, and thereby enforce alter target conformations [28]. Consistent with such putative conformational changes, we observed dramatically altered properties of both the N- and C-terminus of Cobl upon Ca^2+^/CaM signaling. Overall, calcium strongly promoted the actin association with the C-terminus of Cobl in a manner that was independent of CaM association and independent of the first WH2 domain of Cobl. In principle, such Ca^2+^-mediated effects could be due to different mechanisms, i) Ca^2+^ modulates the properties of actin (G-actin or G-actin/F-actin balance), and this is reflected by changed association with Cobl WH2 domains, ii) Ca^2+^ modulates the properties of the Cobl WH2 domains number 2 and 3, and this leads to changed actin affinities or iii) Ca^2+^ signals to further cellular components, and these either modulate actin properties, Cobl properties, or both.

The observed Ca^2+^-mediated increase in Cobl’s overall actin binding occurred despite a simultaneous suppression of the actin binding of the first WH2 domain upon CaM association with neighboring sites, as demonstrated by quantitative coimmunoprecipitation studies and by in vitro reconstitutions with purified components. In line with CaM acting as calcium sensor protein, this suppression was released with decreasing Ca^2+^ levels, whereas the Ca^2+^-promoted actin binding of the Cobl C-terminus persisted. Since all three WH2 domains need to work together and the first WH2 domain is crucial for actin filament formation [12,14], the release of the suppression of the first WH2 domain after a transient Ca^2+^ signal would elegantly ensure that Cobl responds to calcium transients. In contrast to simple on/off mechanisms, transient activations would also exclude Cobl activity during longer lasting NMDA and Ca^2+^/CaM signaling, such as during excitotoxicity conditions. Indeed, such conditions do not promote filament formation but are marked by filament loss [29]. Related to high Ca^2+^ levels during excitotoxicity-reducing actin dynamics and filament formation, high calcium was reported to inhibit the F-actin-driven formation of filopodia-like dendritic spines and stabilized them, whereas lower calcium signals were reported to promote the formation of these F-actin-rich structures. It therefore is conceivable that, besides the crucial role in dendritogenesis, Cobl-mediated functions and Ca^2+^/CaM-mediated control of Cobl may in addition play some role in synapse formation and/or plasticity processes, as these involve both Ca^2+^ signaling and actin cytoskeletal reorganizations [30].

We observed local F-actin accumulations at the base of dendritic protrusions. We have demonstrated earlier that Cobl overexpression promotes dendritic arborization, and Cobl loss-of-function results in a reduction of branches [12,15]. Correlative 3-D-time-lapse studies, inhibitor studies, and mutational analyses demonstrated that dendritic branching is associated with Cobl, F-actin and syndapin I at branch initiation sites and is controlled by changing calcium levels, by Ca^2+^/CaM signaling and by direct Cobl association of CaM.

We have identified a direct plasma membrane association of Cobl that also was CaM-regulated. This ability of the Cobl Homology domain was suppressed upon CaM association. At the same time, Ca^2+^/CaM promoted Cobl’s association with syndapin I [16,31] and thereby promoted indirect membrane associations of Cobl. Direct membrane association of Cobl may ensure its general availability at the plasma membrane and may also explain why syndapin I loss-of-function only partially suppressed Cobl’s cortical localization [16]. Upon CaM binding, syndapin I interactions will increasingly influence Cobl’s localization. Indeed, our studies revealed that dendritic branching events correlated with accumulations of both syndapin I and Cobl.

In line with this conclusion, syndapin I has been demonstrated to be involved in dendritogenesis [16]. Interestingly, we observed accumulations of syndapin I specifically at nascent dendritic branch sites. It thus seems that F-BAR domain-mediated membrane curvature sensing and/or induction by syndapin I [22,32] spatially steers the actin nucleator Cobl at the cell cortex. Consistently, mutational overexpression analyses in neuronal cultures as well as rescue experiments of cerebellar Cobl loss-of-function revealed that CaM binding to the actin-binding C-terminal part and to the syndapin I-binding N-terminal part of Cobl were crucial for Cobl functions in dendritogenesis.

Together, our work unveils that Cobl is regulated by the calcium sensor protein CaM and reveals the Ca^2+^/CaM-controlled molecular mechanisms that are crucial for Cobl’s cellular functions. The regulation by Ca^2+^/CaM seems to distinguish Cobl from established actin nucleators, such as the Arp2/3 complex and Formins, which are directly and indirectly regulated by Rho-type GTPases.

Our examinations of the Ca^2+^/CaM-mediated mechanisms that control Cobl’s activity in neurons furthermore provided deep insights into how local Ca^2+^ signals steer and power branch initiation during early arborization of nerve cells.

## Materials and Methods

### DNA Constructs

Plasmids encoding for GFP-Cobl and deletion mutants thereof were described previously [12,15,16] and generated by PCR and subcloning into pEGFP (Clontech), respectively.

GST fusion proteins of Cobl were generated by subcloning into pGEX-5X-1.

A plasmid encoding for TrxHis-Cobl^54–450^ was generated by PCR and subcloning into a pET32 vector (Novagen).

The yeast-two-hybrid bait used was generated by subcloning Cobl 1001–1337 into pGBTK7 (Clontech).

Plasmids encoding for GFP- and GST-tagged syndapin I full-length and SH3 domain, respectively, were described previously [33,34]. Flag-mCherry-syndapin I was generated using a modified, pCMV-based vector originally expressing Flag-GFP [12]. GST-Abp1 SH3 was described [35].

GST-CaM, TrxHis-CaM, Flag-mCherry-CaM, and Mito-mCherry-CaM were generated by subcloning rat CaM from pCMV-myc-CaM (kindly provided by L. C. Russo [University of São Paulo, São Paulo, Brazil]) into pGEX, pET32, Flag-mCherry, and Mito-targeting vectors [36], respectively.

RNAi constructs directed against mouse Cobl coexpressing GFP and RNAi-insensitive GFP-Cobl full-length (GFP-Cobl*), respectively, as well as the scrambled RNAi vector were described previously [12,15]. RNAi vectors coexpressing GFP-Cobl mutants defective for CaM-binding were generated by combining the generated Cobl mutants with RNAi-resistant parts of Cobl in pRNAT. As a result, using flanking NheI and SmaI sites, GFP-Cobl^*Δ48–229^ (GFP-Cobl^*ΔCaM-N^), GFP-Cobl^*Δ714–1175^ (GFP-Cobl^*ΔCaM-C^), GFP-Cobl^*Δ48–229,714–1175^ (GFP-Cobl^*ΔCaM-N,C^), GFP-Cobl^*Δ1002–1175^ (GFP-Cobl*^ΔCaM-C^’), GFP-Cobl*^Δ1002–1100^ (GFP-Cobl*^ΔCaM-C^”) and GFP-Cobl*^Δ1101–1175^ (GFP-Cobl*^ΔCaM-C^”‘) replaced the GFP reporter.

LifeAct-RFP and-GFP were gifts from K. Murk (Charite Universitätsmedizin Berlin, Germany) described previously [37]. Vectors expressing PM-targeted (farnesylated) mCherry and GFP-GCaMP5G were kindly provided by M. Korte and R. Köster, respectively (both TU Braunschweig, Germany).

Correct cloning by PCR was verified by sequencing in all cases.

### Antibodies and Proteins

Guinea pig and rabbit anti-syndapin I, anti-TrxHis, as well as anti-GST antibodies, were raised and affinity-purified as described previously [34,38]. Guinea pig anti-Cobl antibodies were raised and affinity-purified as described [15]. Polyclonal rabbit anti-GFP antibodies were from Abcam and monoclonal mouse anti-GFP antibodies (B34, JL-8) were from Covance and Clontech, respectively. Monoclonal mouse (M2) and polyclonal rabbit anti-Flag as well as anti-MAP2 (HM-2) antibodies were from Sigma. Polyclonal rabbit anti-CaM antibodies (#4830) were from Cell Signaling Technology. Mouse anti-CaM antibodies (23-132-27) were from DSHB. Monoclonal mouse anti-CaM antibodies (G-3) were from Santa Cruz Biotechnology. Monoclonal mouse and polyclonal rabbit anti-actin antibodies were from Sigma. Polyclonal rabbit anti-MAP2 antibodies were from Abcam. Phalloidin AlexaFluor^®^488 and MitoTracker were from Molecular Probes.

Secondary antibodies used included, Alexa Fluor488- and 568-labeled goat anti-guinea pig antibodies, Alexa Fluor488- and 568-labeled donkey anti-mouse as well as Alexa Fluor647-labeled goat anti-mouse antibodies, Alexa Fluor488-labeled donkey anti-rabbit, Alexa Fluor568- and 647-labeled goat anti-rabbit antibodies and AlexaFluor680-labeled goat anti-rabbit and anti-mouse antibodies (Molecular Probes); goat anti-rabbit, anti-guinea pig, and anti-mouse-peroxidase antibodies (Dianova); DyLight800-conjugated goat anti-rabbit and anti-mouse antibodies (Pierce) and donkey anti-guinea pig antibodies coupled to IRDye680 and IRDye800, respectively, (LI-COR Bioscience).

Rabbit skeletal muscle actin was from Cytoskeleton. GST- and TrxHis-tagged fusion proteins were purified from *E*. *coli* as described previously [16,39].

### In Vitro Reconstitutions of Direct Protein–Protein and Protein–Liposome Interactions

Direct protein–protein interactions were demonstrated by coprecipitations with combinations of recombinant TrxHis- and GST-tagged fusion proteins purified from *E*. *coli* and CaM-sepharose 4B (GE Healthcare), respectively. TrxHis-CaM regulation of GST-Cobl^1001–1224^/actin complex formation was demonstrated in 10 mM HEPES pH 7.4, 0.1 mM MgCl_2_, 1% (v/v) Triton X-100 (EGTA-free lysis buffer) with 250 mM NaCl, 500 μM Ca^2+^, 0.2 mM ATP, and 0.5 mM DTT supplemented with EDTA-free protease inhibitor cocktail and 200 μM calpain inhibitor I (Sigma).

Studies of TrxHis-Cobl^54–450^/GST-syndapin I/GST-CaM complex formation and controls were done in EGTA-free lysis buffer with EDTA-free protease inhibitor cocktail, 200 mM NaCl, and 500 μM Ca^2+^.

Liposome-binding assays were conducted with lipids from Folch-fraction type I (Sigma) essentially as described previously [22,40].

Analyses addressing direct interactions of GST-Cobl^1001-1176^ with CaM were done in lysis buffer with 150 mM NaCl containing 0 and 1 μM Ca^2+^, respectively (set according to [41]). Eluted proteins were analyzed by SDS-PAGE and subsequent anti-TrxHis and anti-GST immunoblotting.

### Preparation of HEK293 Cell Lysates

24–48 h after transfection, HEK293 cells were washed with PBS, harvested and subjected to sonification for 10 s and/or lysed by incubation in lysis buffer containing EDTA-free protease inhibitor Complete (Roche) and 120–150 mM NaCl for 20 to 30 min at 4°C. Cell lysates were obtained as supernatants from centrifugations at 16,000 xg (20 min at 4°C).

### Coprecipitation of Proteins from HEK293 Cell Lysates

For coprecipitation experiments, extracts from HEK293 cells expressing different GFP fusion proteins were incubated for 3 h at 4°C with purified GST-fusion proteins immobilized on glutathione sepharose beads (GenScript) as described [33]. Bound protein complexes were eluted with 20 mM-reduced glutathione, 120 mM NaCl, 50 mM Tris/HCl pH 8.0.

For coprecipitations with CaM, HEK293 cell lysates were prepared in EGTA-free lysis buffer containing 150 mM NaCl, and EDTA-free protease inhibitor cocktail and 200 μM calpain inhibitor I. Cell lysates were supplemented with either 1 mM EGTA or to be tested Ca^2+^ concentrations.

For binding curves, Ca^2+^ concentrations ranging from 0 to 500 μM were set according to [41]. After incubation with 25 μl CaM-sepharose 4B for 3 h at 4°C and washing, bound proteins were isolated by boiling in SDS sample buffer.

Lysates, supernatants, and eluates were analyzed by immunoblotting using anti-GST and anti-GFP antibodies, respectively.

For binding curves, quantitative immunoblotting experiments (n ≥ 3) were conducted and data expressed as percent binding, %(Elution/∑Elution)/(Elution/∑Elution+Supernatant/∑Supernatant) with pEGFP control values subtracted. Sigmoidal dose-response curves were fit from Graphpad Prism and modified using Adobe Illustrator.

### Coimmunoprecipitation Analyses

Lysates from HEK293 cells transfected with GFP-Cobl^106–324^ or GFP together with Flag-mCherry (FlagC)-tagged CaM or Flag-mCherry were incubated with 2 μg of rabbit anti-Flag antibody and nonimmune rabbit IgG (Santa Cruz Biotechnology) prebound (3 h, 4°C) to protein A/G-agarose (Santa Cruz Biotechnology) in lysis buffer containing 50 mM NaCl.

Coimmunoprecipitations of endogenous actin together with GFP and GFP-tagged Cobl deletion mutants were done according to [12] with slight modifications. Lysates of HEK293 cell were incubated in EGTA-free lysis buffer containing 100 mM NaCl, 5 mM DTT, 200 μM calpain inhibitor I and varying amounts (2 μM, 500 μM) of Ca^2+^ or no free Ca^2+^ (EGTA addition) with 5 μg rabbit anti-GFP antibody/well (6-well plate) for 3 h at 4°C.

Reversibility of Ca^2+^-induced suppression of actin binding of Cobl^1001–1224^ by CaM association was tested by incubating 2 μM Ca^2+^-treated samples subsequently with 1 mM EGTA for 2 h. The CaM-independent Ca^2+^-induced (500 μM Ca^2+^) increase of actin binding to Cobl’s C-terminal part (Cobl^1176–1337^) was tested for reversibility by subsequent EGTA addition (5 mM EGTA final). Antibody-associated protein complexes were isolated with protein A/G-agarose (2 h, 4°C), washed with a buffer with the respective Ca^2+^ concentrations and eluted by boiling in a mix of 8 M urea and 4x-SDS-sample buffer. The eluates were immunoblotted with anti-GFP and anti-actin antibodies and analyzed quantitatively using fluorescently labeled secondary antibodies and a LI-COR Odyssey System.

Quantitative coimmunoprecipitation analyses of Flag-syndapin I with GFP-Cobl proteins were done similarly except that the lysis buffer lacked DTT and contained 75 mM NaCl and protein A-agarose (Santa Cruz Biotechnology) was used. Confirmatory experiments were additionally done at 100 mM NaCl for consistency with previously published conditions for heterologous syndapin I/Cobl coimmunoprecipitations [16].

Coimmunoprecipitations of endogenous Cobl and CaM were performed using rat brain lysates in lysis buffer with 30 mM NaCl, 500 μM Ca^2+^ and 200 μM calpain inhibitor I using mouse anti-CaM (G-3) antibodies according to coimmunoprecipitation procedures described [16]. Coimmunoprecipitations of endogenous Cobl and syndapin I were performed using rat brain lysates in lysis buffer with 30 mM NaCl and 400 μM calpain inhibitor I with and without 2 μM Ca^2+^ using guinea pig anti-Cobl^DBY^ antibodies as described [16], except that antibodies were added to brain lysates and then isolated with protein A-agarose.

The amounts of coimmunoprecipitated proteins under different conditions were normalized to the amount of immunoprecipitated GFP-Cobl and Cobl, respectively, and expressed as percent difference from Ca^2+^-free conditions. Statistical analyses were performed using one-way ANOVA with Tukey’s post-test. *p < 0.05, **p < 0.01, ***p < 0.001.

### Characterization of Anti-CaM Antibodies

Lysates from HEK293 cells transfected with GFP-CaM were incubated with 5 μg rabbit anti-GFP antibodies, 10 μl rabbit anti-CaM antibodies (4830, Cell Signaling), 2 μg mouse anti-CaM (G-3, Santa Cruz) antibodies and 50 μl mouse anti-CaM (23-132-27 DSHR) antibodies, respectively, for 3 h, 4°C in EGTA-free lysis buffer containing 30 mM NaCl, 0.5 mM Ca^2+^ EDTA-free protease inhibitor cocktail and calpain inhibitor I and thereafter for 2 h with protein A/G-agarose (4°C). 5 μg rabbit and mouse anti-IgG was used as specificity controls. Immunoprecipitated material was analyzed by immunoblotting with anti-GFP antibodies and the anti-CaM antibodies, respectively.

### Cell culture, Transfection, and Immunostaining

Culturing of HEK293 and COS-7 cells and immunolabeling were essentially as described [42]. HEK293 and COS-7 cells were transfected using TurboFect (Thermo Scientific). Mitochondria of COS-7 cells were stained with 0.2 μM MitoTracker Deep Red FM (Molecular Probes) in medium at 37°C for 1 h and cells were subsequently fixed with 4% PFA for 7 min.

Primary rat hippocampal neuron cultures for immunofluorescence analyses were prepared, maintained, and transfected (at DIV4) as described previously [16,43,44]. Fixation was done at DIV6 in 4% (w/v) PFA in PBS pH 7.4 at RT for 4–6 min. Permeabilization and blocking were done with 10% (v/v) horse serum, 5% (w/v) BSA in PBS with 0.2% (v/v) Triton X-100. Phalloidin stainings and antibody incubations were done in the same buffer without Triton X-100 according to [42,44].

### Immunolabeling of Mouse Brain Sections

Brain sections of adult (7 weeks) male mice were prepared and immunolabeled as described [15].

### RT-PCR from Murine Brain

Brain preparations at different developmental stages, mRNA and cDNA preparation, as well as RT-PCR, were done according to [15] using the primers GCTCCGGAAGACTGCAGAACA (forward-WH2; positioned at exon border 12/13) and CGAGCAAGGGAACCTTTCTTAGTC (reverse-WH2; positioned at exon border 14/15) for Cobl detection and the primers ATTGACCTCAACTACATGGTCTACA (forward) and CCAGTAGACTCCACGACATACTC (reverse) for GAPDH as control.

### Microscopy

Confocal images were recorded using a Leica TCS SP5 microscope (equipped with 40x/0.75dry and 63x/1.4 oil objectives, LAS AF software), a Zeiss LSM Meta 510 (using the 20x/0.5dry objective and ZEN software) or a Zeiss AxioObserver.Z1 microscope equipped with an ApoTome. Both Zeiss microscopes were equipped with Plan-Apochromat 100x/1.4, 63x/1.4, 40x/1.3 and 20x/0.5 objectives and an AxioCam MRm CCD camera (Zeiss). Digital images from Zeiss microscopes were recorded by ZEN2012 or AxioVision Software (Vs40 4.8.2.0). Image processing was done by Adobe Photoshop.

### Spinning Disc Live Microscopy of Developing Neurons

Primary hippocampal neurons undergoing dendritic arbor formation were transiently transfected with Lipofectamine 2000 at DIV6. For imaging, the culture medium was replaced by 20 mM HEPES pH 7.4, 0.8 mM MgCl_2_, 1.8 mM CaCl_2_, 5 mM KCl, 140 mM NaCl, 5 mM D-glucose (live imaging buffer) adjusted to isoosmolarity using a freezing point osmometer (Osmomat 3000; Gonotec). For inhibitor studies, 10 μM (final) CaM inhibitor CGS9343B (Tocris) was used in DMSO and accompanied with the respective solvent control (0.1% DMSO final). Live imaging was conducted 16–28 h after transfection in an open coverslip holder placed into a temperature- and CO_2_-controlled incubator built around a spinning disc microscope. The microscope was a motorized Axio Observer combined with a spinning disc unit CSU-X1A 5000 and equipped with a 488 nm/100 mW OPSL laser, a 561 nm/40 mW diode laser and a QuantEM 512SC EMCCD camera (Zeiss). Images were taken as Z-stacks (stacks of 7–17 images at Z-intervals of 0.31 μm depending on cellular morphology) at time intervals of 10 s and 3 s (Ca^2+^ imaging) with exposure times of 50–200 ms and 3%–12% laser power using a C-Apochromat objective (63x/1.20W Korr M27; Zeiss).

Image processing was done using ZEN, Imaris software, and Adobe Photoshop.

Quantitative comparisons of signal intensities at dendritic branch initiation sites with signal intensities at dendritic sites that were not branching were done by placing a circular ROI at the branch initiation site (covering the dendrite diameter) and on an adjacent dendrite section (distance 2 ROI diameters). The fluorescence signal intensities of GFP and GFP-Cobl were measured at both sites 30 s prior to initiation of protrusion and expressed as relative enrichments relative to control ROI.

Frequencies of protrusion initiation from dendrite sections of neurons incubated with DMSO control and CaM inhibitor CGS9343B in DMSO were compared to those prior to treatment and expressed as protrusions per 10 μm dendrite section and min.

### Quantitative Analyses of Dendrites of Hippocampal Neurons in Culture and of Purkinje Cells in Cerebellar Slice Cultures

Dendrite analyses of transiently transfected (DIV4) hippocampal neurons immunostained for MAP2 (NM_013066.1; GI:6981181) were performed with ≥2 independent neuronal preparations on 2–6 independent coverslips per condition in each assay at DIV6. Neurons were sampled systematically on each coverslip. Morphometric measurements were based on anti-MAP2 immunolabeling and performed with ImageJ according to [16,44]. The number of dendrites and the number of dendritic branching points were determined from 53 to 197 neurons (DIV6) for each condition. The CaM inhibitors W7 (N-(6-Aminohexyl)-5-chlor-1-naphthalinsulfonamide; Tocris) and CGS9343B (1,3-dihydro-l-[1-[4-methy1-4H,6H-pyrrolo[1,2-a][4,l]-benzoxazepin-4-y1-methy1]-4-piperidinyl]-2H-benzimidazol-2-one(1:1) maleate; Tocris) were applied 30 h after transfection at final concentrations of 10 μM and 0.1% DMSO for 18 h. All data were normalized to internal GFP controls run in parallel in each individual experiment and neuronal preparation.

Preparation of cerebellar slices, gene gun transfection and morphometric analyses of Purkinje cell dendrites in the Molecular Layer of the cerebellum were done as described [15].

Statistical analyses were performed using GraphPad Prism 5 and 6, respectively, and one-way ANOVA with Tukey’s post-test. *p < 0.05, **p < 0.01, ***p < 0.001.

## Supporting Information

S1 Data Numerical information underlying all quantitative analyses presented in the main and the supplementary figures in the Supporting Information. S1 Data is an Excel file containing all data underlying Fig 1D; Fig 4G, 4H and 4J; Fig 5E, 5F and 5H–5J; Fig 8B, 8C and 8E; Fig 10K, 10L and 10V; S6D–S6G Fig; S8D and S8E Fig as separate tabs.
(XLSX)

S1 Fig 3-D-time-lapse analyses of the dynamics of GFP-Cobl during dendritic branch induction. Original maximum intensity projections (MIPs) of the 3-D-time-lapse recording shown as heat map representations in Fig 1C. GFP-Cobl is dynamically enriched at distinct sites within dendrites in immature neurons undergoing dendritogenesis. Initiation of dynamic, dendritic protrusions (marked by green *) often is preceded by Cobl accumulation (white arrows). Retraction events are marked by red ° and static phases with yellow I. Dendrite branch induction is a dynamic process with often several protrusive attempts until a dendritic branch is firmly established and strongly elongated. Three consecutive initiations of a protrusion from the same site are shown. Bar, 5 μm. Please also see S1 Movie.
(TIF)

S2 Fig Characterization of the Mito-mCherry-CaM-based recruitment assay for the analysis of CaM binding partners in intact cells. (**A**) COS-7 cells transfected with Mito-mCherry-CaM show a successful targeting of Mito-mCherry-CaM to mitochondria stained with MitoTracker. (**B**) Negative control demonstrating that GFP is not recruited to CaM-coated mitochondria. (**C,D**) Specificity control experiments showing that a related mitochondrially targeted fluorescent protein lacking CaM (Mito-mCherry) is unable to recruit Cobl proteins. Bars in **A–D**, 10 μm.
(TIF)

S3 Fig Characterization of anti-CaM antibodies for their use in immunoprecipitation and immunoblotting. (**A**) Immunoprecipitation of rat GFP-CaM with anti-GFP antibodies and three commercial anti-CaM antibodies, respectively. Immunoprecipitated material was detected by immunoblotting with anti-GFP antibodies and specificities of immunoprecipitations were controlled for by using IgGs of the respective species. Note that only anti-GFP (positive control) and anti-CaM G-3 antibodies were able to immunoprecipitate GFP-CaM. (**B**) Immunoblotting analyses of immunoprecipitated GFP-CaM with the different antibodies. Besides anti-GFP antibodies (positive control), also anti-CaM G3 and anti-CaM 23-132-27 antibodies were able to recognize GFP-CaM.
(TIF)

S4 Fig Correlation of Cobl and CaM dynamics at forming dendritic protrusions. MIPs from 3-D-time-lapse recordings of GFP-Cobl and mCherry-CaM in a dendrite of a primary hippocampal neuron transfected at DIV6 and imaged at DIV7 show that episodes of Cobl accumulation at distinct dendritic sites as well as the induction of protrusions from such sites are accompanied by accumulations of CaM at the same sites. For heat map representation of the individual channels showing GFP-Cobl and mCherry-CaM see Fig 3A. Bar, 2 μm. Please also see S3 Movie.
(TIF)

S5 Fig RT-PCR analyses of the expression of Cobl during different stages of murine brain development. Images of agarose gels (inverted) with amplifications of the Cobl cDNA. GAPDH served as positive control. Reactions without reverse transcriptase (no Rev. Tr.) served as negative controls.
(TIF)

S6 Fig CaM inhibition suppresses dendritic branching during the development of Purkinje cells in cerebellar slice cultures. (**A–C**) Parasagittal cerebellar slices (250 μm) of postnatal day 10 (P10) mice cultured for 2 d showing individual Purkinje cells transfected with a GFP-expressing reporter plasmid and incubated with the indicated CaM inhibitors W7 and CGS9343B as well as with DMSO (solvent control), respectively. DAPI in Blue. Bar, 20 μm. (**D–G**) Quantification of morphometric parameters of Purkinje cell arborization in cerebellar slice cultures. Data are mean ± SEM. DMSO, *n* = 6; W7, *n* = 5; CGS9343B, *n* = 7 cells. For data underlying **D–G,** see S1 Data. Statistical significances were tested using one-way ANOVA with Tukey’s post-test. *p < 0.05, **p < 0.01, ***p < 0.001.
(TIF)

S7 Fig Reconstitutions of GFP-Cobl^750-1005^/CaM and GFP-Cobl^1001-1176^/CaM complexes in intact COS-7 cells. (**A–D**) Visualization of the Cobl/CaM interaction in intact COS-7 cells by recruitment of GFP-Cobl deletion mutants (Cobl^750-1005^ and Cobl^1001-1176^) to mitochondrially anchored mCherry-CaM (Mito-mCherry-CaM) (**A,B**) but not to Mito-mCherry (**C,D**). Bars, 10 μm. For further controls see the Supporting Information (S2 Fig).
(TIF)

S8 Fig CaM associates with the Cobl N- and C-terminus in a Ca^2+^ concentration-dependent manner. (**A–E**) Quantitative coprecipitation analyses of GFP-Cobl deletion mutants containing the CaM binding sites within the N-terminal Cobl Homology domain (Cobl^106–324^) (**A**) and the CaM binding site located close to the C-terminal WH2 domains (Cobl^1001–1337^) (**B**), as well as of a GFP control (**C**) with immobilized CaM under different calcium concentrations. Note that both Cobl fusion proteins bind to CaM in a specific manner (**A–C**) and that quantitative Western blotting analyses (**D,E**) show that half-maximal binding is already reached at 0.68 μM and 0.95 μM Ca^2+^, respectively. Please also note that 2 μM Ca^2+^ used in some biochemical assays of this study corresponds to about 80%–90% of maximal binding observed and that 500 μM Ca^2+^ ensures plateau levels of CaM association. GFP-Cobl^106–324^, *n* = 4; GFP-Cobl^1001–1337^, *n* = 7; GFP, *n* = 3. For data underlying **D** and **E** see S1 Data.
(TIF)

S9 Fig The interaction of Cobl^1001-1176^ with CaM is Ca^2+^-dependent and direct. (**A–D**) In vitro reconstitution experiments using immobilized CaM and purified GST-Cobl^1001-1176^ (**A,B**) and a GST control, respectively (**C,D**), show that the Cobl/CaM interaction is direct and Ca^2+^-dependent.
(TIF)

S10 Fig Actin association by a combination of first WH2 domain and CaM interface is inhibited upon Ca^2+^ addition. Immunoblottings of immunoprecipitations of GFP-Cobl^1176-1224^, GFP-Cobl^1001–1224^, and GFP-Cobl^1001-1176^ with anti-GFP antibodies from HEK293 lysates (IP, anti-GFP) show that specific coimmunoprecipitation of endogenous actin (CoIP, anti-actin) requires a combination of the first WH2 domain and the CaM binding interface and demonstrate that this actin association of the first WH2 domain of Cobl is inhibited by Ca^2+^ addition (red arrowhead).
(TIF)

S11 Fig Input of purified proteins used for the in vitro reconstitution of the Ca^2+^/CaM-mediated suppression of the actin binding of the first WH2 domain of Cobl. Immunoblot analysis of the input of purified proteins used for the in vitro reconstitution of the Ca^2+^/CaM-mediated suppression of the actin binding of the first WH2 domain of Cobl (see Fig 5I) by a mix of anti-TrxHis, anti-GST, and anti-actin antibodies.
(TIF)

S12 Fig Confirmation of the promotion of the syndapin I interaction of the Cobl Homology domain under alternative conditions of immunoprecipitation. Coimmunoprecipitations of GFP-Cobl^1-408^/Flag-syndapin I (Flag-Sdp I) along with a corresponding GFP control from HEK293 cells under Ca^2+^-free conditions (−) and 2 μM Ca^2+^ (+). Note that the syndapin I interaction with Cobl is promoted upon increasing the Ca^2+^ concentration (indicated by the green upright arrowhead). The data shown corroborate the quantitative coimmunoprecipitation analyses shown in Fig 8A–8C under increased salt conditions (100 mM NaCl).
(TIF)

S13 Fig Syndapin I and Cobl both accumulate at sites of dendritic branching prior to branch induction. Time-lapse spinning disc microscopy images (MIPs) of a dendritic protrusion (*) emanating from a Cobl and syndapin I-enriched site (arrows). For heat map representations of the data for GFP-Cobl and Flag-mCherry-syndapin I, see Fig 9B. Bar, 5 μm.
(TIF)

S14 Fig The C terminus of Cobl contains at least two CaM binding sites. Coprecipitation experiments with immobilized CaM and GFP-Cobl deletion mutants in presence and absence of 500 μM Ca^2+^ identified two independent sites within Cobl^1001-1176^ that specifically associated with CaM. GFP fusion proteins expressed (lysates) and coprecipitated with CaM, respectively, were detected by immunoblotting with anti-GFP antibodies.
(TIF)

S15 Fig A Cobl mutant deficient for N-terminal CaM binding still associates with syndapin I and Abp1. (**A,B**) Coprecipitation experiments demonstrating that both Flag-GFP-Cobl^1-713^ and the corresponding CaM binding-deficient mutant Flag-GFP-Cobl^1-713∆48–229^ specifically associate with immobilized GST-SH3 domain fusion proteins of two components crucial for Cobl’s functions in dendritogenesis, syndapin I (**A**) and Abp1 (**B**).
(TIF)

S1 Movie 3-D-time-lapse movie of GFP-Cobl corresponding to Fig 1C. 3-D-time-lapse movie of GFP-Cobl in dendrite sections of a primary hippocampal neuron transfected at DIV6 and imaged at DIV7 corresponding to Fig 1C. Cobl is dynamically enriched at distinct sites within dendrites. Initiation of dynamic, dendritic protrusions is commonly marked by preceding Cobl accumulation. Bar, 1 μm.
(AVI)

S2 Movie 3-D-time-lapse movie of GFP-Cobl and LifeAct-RFP corresponding to Fig 1E. 3-D-time-lapse movie of GFP-Cobl and LifeAct-RFP in a dendrite section of a primary hippocampal neuron transfected at DIV6 and imaged at DIV7 corresponding to Fig 1E. Note that Cobl and F-actin accumulate at the same sites within the dendritic arbor and that the F-actin build-up coinciding with the initiation of dendritic protrusions follows Cobl accumulation at the dendritic base. Bar, 0.7 μm.
(AVI)

S3 Movie 3-D-time-lapse movie of GFP-Cobl and mCherry-CaM corresponding to Fig 3A. 3-D-time-lapse movie of GFP-Cobl and mCherry-CaM dynamics in a dendrite section of a primary hippocampal neuron transfected at DIV6 and imaged at DIV7 corresponding to Fig 3A. Episodes of spatial accumulation of Cobl at distinct dendritic sites as well as the induction of protrusions from such sites overlap with accumulations of CaM. Bar, 0.8 μm.
(AVI)

S4 Movie 3-D-time-lapse imaging of GFP-Cobl dynamics and PM-mCherry before and after incubation with CaM inhibitor GCS9343B corresponding to Fig 4I. 3-D-time-lapse imaging of GFP-Cobl dynamics and of neuronal morphogenesis visualized by PM-mCherry in a primary hippocampal neuron transfected at DIV6 and imaged at DIV7 before and after incubation with CaM inhibitor GCS9343B corresponding to selected images shown in Fig 4I. Most neuronal structures are dynamic but GCS9343B addition (at 11:20 min:s; marked by 1 frame with red signal only) impeded these morphological dynamics and neuronal structures largely were static until the end of recording. Bar, 2 μm.
(AVI)

## Abbreviations

CaM: calmodulin
CaMK: CaM kinase
DIV: days in vitro
EGTA: ethylenglycol-bis(aminoethylether)-N,N,N′,N′-tetraacetic acid
MIP: maximum intensity projection
NMDA: N-methyl-D-aspartic acid
P10: postnatal day 10
ROI: region of interest
WASP: Wiskott-Aldrich syndrome protein
WH2: WASP Homology 2

## References

[1] Rajan I, Cline HT. Glutamate receptor activity is required for normal development of tectal cell dendrites in vivo. J Neurosci. 1998;18: 7836–7846. 9742152 10.1523/JNEUROSCI.18-19-07836.1998 PMC6793000

[2] Chevaleyre V, Castillo PE. Assessing the role of Ih channels in synaptic transmission and mossy fiber LTP. Proc Natl Acad Sci U S A. 2002;99: 9538–9543. 12093909 10.1073/pnas.142213199 PMC123176

[3] Ohashi R, Sakata S, Naito A, Hirashima N, Tanaka M. Dendritic differentiation of cerebellar Purkinje cells is promoted by ryanodine receptors expressed by Purkinje and granule cells. Dev Neurobiol. 2014;74: 467–480. 10.1002/dneu.22139 24123915

[4] Gaudillière B, Konishi Y, de la Iglesia N, Yao G, Bonni A. A CaMKII-NeuroD signaling pathway specifies dendritic morphogenesis. Neuron. 2004;41: 229–241. 14741104 10.1016/s0896-6273(03)00841-9

[5] Fink CC, Bayer KU, Myers JW, Ferrell JE Jr, Schulman H, Meyer T. Selective regulation of neurite extension and synapse formation by the β but not the α isoform of CaMKII. Neuron. 2003;39: 283–297. 12873385 10.1016/s0896-6273(03)00428-8

[6] Rohatgi R, Ma L, Miki H, Lopez M, Kirchhausen T, Takenawa T, et al The interaction between N-WASP and the Arp2/3 complex links Cdc42-dependent signals to actin assembly. Cell. 1999;97: 221–231. 10219243 10.1016/s0092-8674(00)80732-1

[7] Miki H, Yamaguchi H, Suetsugu S, Takenawa T. IRSp53 is an essential intermediate between Rac and WAVE in the regulation of membrane ruffling. Nature. 2000;408: 732–735. 11130076 10.1038/35047107

[8] Li F, Higgs HN. The mouse Formin mDia1 is a potent actin nucleation factor regulated by autoinhibition. Curr Biol. 2003;13: 1335–1340. 12906795 10.1016/s0960-9822(03)00540-2

[9] Lammers M, Rose R, Scrima A, Wittinghofer A. The regulation of mDia1 by autoinhibition and its release by Rho*GTP. EMBO J. 2005; 24: 4176–4187. 16292343 10.1038/sj.emboj.7600879 PMC1356318

[10] Qualmann B, Kessels MM. New players in actin polymerization–WH2-domain-containing actin nucleators. Trends Cell Biol. 2009;19: 276–285. 10.1016/j.tcb.2009.03.004 19406642

[11] Campellone KG, Welch MD. A nucleator arms race: cellular control of actin assembly. Nat Rev Mol Cell Biol. 2010;11: 237–251. 10.1038/nrm2867 20237478 PMC2929822

[12] Ahuja R, Pinyol R, Reichenbach N, Custer L, Klingensmith J, Kessels MM, et al Cordon-bleu is an actin nucleation factor and controls neuronal morphology. Cell. 2007;131: 337–350. 17956734 10.1016/j.cell.2007.08.030 PMC2507594

[13] Firat-Karalar EN, Hsiue PP, Welch MD. The actin nucleation factor JMY is a negative regulator of neuritogenesis. Mol Biol Cell. 2011;22: 4563–4574. 10.1091/mbc.E11-06-0585 21965285 PMC3226475

[14] Husson C, Renault L, Didry D, Pantaloni D, Carlier MF. Cordon-Bleu uses WH2 domains as multifunctional dynamizers of actin filament assembly. Mol Cell. 2011;43: 464–477. 10.1016/j.molcel.2011.07.010 21816349

[15] Haag N, Schwintzer L, Ahuja R, Koch N, Grimm J, Heuer H, et al The actin nucleator Cobl is crucial for Purkinje cell development and works in close conjunction with the F-actin binding protein Abp1. J Neurosci. 2012;32: 17842–17856. 10.1523/JNEUROSCI.0843-12.2012 23223303 PMC6621670

[16] Schwintzer L, Koch N, Ahuja R, Grimm J, Kessels MM, Qualmann B. The functions of the actin nucleator Cobl in cellular morphogenesis critically depend on syndapin I. EMBO J. 2011;30: 3147–3159. 10.1038/emboj.2011.207 21725280 PMC3160182

[17] Sutoo D, Akiyama K, Fujii N, Matsushita K. H-NMR studies of calmodulin: the effect of W-7 (N-(6-aminohexyl)-5-chloro-1-naphthalenesulfonamide) and Ca^2+^ on conformational changes of calmodulin. Biochim Biophys Acta. 1986;873: 156–160. 3741880 10.1016/0167-4838(86)90203-7

[18] Norman JA, Ansell J, Stone GA, Wennogle LP, Wasley JWF. CGS 9343B, a novel, potent, and selective inhibitor of calmodulin activity. Mol Pharmacol. 1987;31: 535–540. 3033469

[19] Minowa O, Yagi K. Calcium binding to tryptic fragments of calmodulin. J Biochem. 1984;96: 1175–1182. 6520119 10.1093/oxfordjournals.jbchem.a134935

[20] Shifman JM, Choi MH, Mihalas S, Mayo SL, Kennedy MB. Ca^2+^/calmodulin-dependent protein kinase II (CaMKII) is activated by calmodulin with two bound calciums. Proc Natl Acad Sci U S A. 2006;103: 13968–13973. 16966599 10.1073/pnas.0606433103 PMC1599897

[21] Valeyev NV, Bates DG, Heslop-Harrison P, Postlethwaite I, Kotov NV. Elucidating the mechanisms of cooperative calcium-calmodulin interactions: a structural systems biology approach. BMC Syst Biol. 2008;2: 48–65. 10.1186/1752-0509-2-48 18518982 PMC2435525

[22] Dharmalingam E, Haeckel A, Pinyol R, Schwintzer L, Koch D, Kessels MM, et al F-BAR proteins of the syndapin family shape the plasma membrane and are crucial for neuromorphogenesis. J Neurosci. 2009;29: 13315–13327. 10.1523/JNEUROSCI.3973-09.2009 19846719 PMC6665186

[23] Schneider K, Seemann E, Liebmann L, Ahuja R, Koch D, Westermann M, et al ProSAP1 and membrane nanodomain-associated syndapin I promote postsynapse formation and function. J Cell Biol. 2014;205: 197–215. 10.1083/jcb.201307088 24751538 PMC4003247

[24] Lohmann C. Calcium signaling and the development of specific neuronal connections. Prog Brain Res. 2009;175: 443–452. 10.1016/S0079-6123(09)17529-5 19660672

[25] Riederer BM, Zagon IS, Goodman SR. Brain spectrin(240/235) and brain spectrin(240/235E): two distinct spectrin subtypes with different locations within mammalian neural cells. J Cell Biol. 1986;102: 2088–2097. 3519621 10.1083/jcb.102.6.2088 PMC2114251

[26] Wyszynski M, Lin J, Rao A, Nigh E, Beggs AH, Craig AM, et al Competitive binding of α-actinin and calmodulin to the NMDA receptor. Nature. 1997;385: 439–442. 9009191 10.1038/385439a0

[27] Hall DD, Dai S, Tseng PY, Malik Z, Nguyen M, Matt L, et al Competition between α-actinin and Ca^2+^-calmodulin controls surface retention of the L-type Ca^2+^ channel Ca_V_1.2. Neuron. 2013;78: 483–497. 10.1016/j.neuron.2013.02.032 23664615 PMC4570828

[28] Hudmon A, Schulman H. Neuronal Ca^2+^/calmodulin-dependent protein kinase II: the role of structure and autoregulation in cellular function. Annu Rev Biochem. 2002;71: 473–510. 12045104 10.1146/annurev.biochem.71.110601.135410

[29] Smart FM, Halpain S. Regulation of dendritic spine stability. Hippocampus. 2000;10: 542–554. 11075824 10.1002/1098-1063(2000)10:5<542::AID-HIPO4>3.0.CO;2-7

[30] Lohmann C, Finski A, Bonhoeffer T. Local calcium transients regulate the spontaneous motility of dendritic filopodia. Nat Neurosci. 2005; 8: 305–312. 15711541 10.1038/nn1406

[31] Schüler S, Hauptmann J, Perner B, Kessels MM, Englert C, Qualmann B. Ciliated sensory hair cell formation and function require the F-BAR protein syndapin I and the WH2 domain-based actin nucleator Cobl. J Cell Sci. 2013;126: 196–208. 10.1242/jcs.111674 23203810

[32] Qualmann B, Koch D, Kessels MM. Let's go bananas: revisiting the endocytic BAR code. EMBO J. 2011;30: 3501–3515. 10.1038/emboj.2011.266 21878992 PMC3181480

[33] Kessels MM, Qualmann B. Syndapin oligomers interconnect the machineries for endocytic vesicle formation and actin polymerization. J Biol Chem. 2006;281: 13285–13299. 16540475 10.1074/jbc.M510226200

[34] Qualmann B, Roos J, DiGregorio PJ, Kelly RB. Syndapin I, a synaptic dynamin-binding protein that associates with the neural Wiskott-Aldrich syndrome protein. Mol Biol Cell. 1999;10: 501–513. 9950691 10.1091/mbc.10.2.501 PMC25183

[35] Kessels MM, Engqvist-Goldstein ÅEY, Drubin DG. Association of mouse actin-binding protein 1 (mAbp1/SH3P7), an Src kinase target, with dynamic regions of the cortical actin cytoskeleton in response to Rac1 activation. Mol Biol Cell. 2000;11: 393–412. 10637315 10.1091/mbc.11.1.393 PMC14781

[36] Kessels MM, Qualmann B. Syndapins integrate N-WASP in receptor-mediated endocytosis. EMBO J. 2002;21: 6083–6094. 12426380 10.1093/emboj/cdf604 PMC137196

[37] Riedl J, Crevenna AH, Kessenbrock K, Yu JH, Neukirchen D, Bista M, et al Lifeact: a versatile marker to visualize F-actin. Nat Methods. 2008;5: 605–607. 10.1038/nmeth.1220 18536722 PMC2814344

[38] Braun A, Pinyol R, Dahlhaus R, Koch D, Fonarev P, Grant B.D, et al EHD proteins associate with syndapin I and II and such interactions play a crucial role in endosomal recycling. Mol Biol Cell. 2005;16: 3642–3658. 15930129 10.1091/mbc.E05-01-0076 PMC1182304

[39] Qualmann B, Kelly RB. Syndapin isoforms participate in receptor-mediated endocytosis and actin organization. J Cell Biol. 2000;148: 1047–1062. 10704453 10.1083/jcb.148.5.1047 PMC2174535

[40] Koch N, Dharmalingam E, Westermann M, Qualmann B, Thomas U, Kessels MM. Abp1 utilizes the Arp2/3 complex activator Scar/WAVE in bristle development. J Cell Sci. 2012;125: 3578–3589. 22467854 10.1242/jcs.101451

[41] Bers DM, Patton CW, Nuccitelli R. A practical guide to the preparation of Ca^2+^ buffers. Methods Cell Biol. 1994;40: 3–29. 8201981 10.1016/s0091-679x(08)61108-5

[42] Kessels MM, Engqvist-Goldstein ÅEY, Drubin DG, Qualmann B. Mammalian Abp1, a signal-responsive F-actin-binding protein, links the actin cytoskeleton to endocytosis via the GTPase dynamin. J. Cell Biol. 2001;153: 351–366. 11309416 10.1083/jcb.153.2.351 PMC2169459

[43] Qualmann B, Boeckers TM, Jeromin M, Gundelfinger ED, Kessels MM. Linkage of the actin cytoskeleton to the postsynaptic density via direct interactions of Abp1 with the ProSAP/Shank family. J Neurosci. 2004;24: 2481–2495. 15014124 10.1523/JNEUROSCI.5479-03.2004 PMC6729500

[44] Pinyol R, Haeckel A, Ritter A, Qualmann B, Kessels MM. Regulation of N-WASP and the Arp2/3 complex by Abp1 controls neuronal morphology. PLoS One. 2007;2: e400 17476322 10.1371/journal.pone.0000400 PMC1852583

