# Supplementary figures and images for: The Actin Nucleator Cobl Is Controlled by Calcium and Calmodulin
Source: PLoS Biol. 2015 Sep 3;13(9):e1002233. doi: 10.1371/journal.pbio.1002233 (PMC4559358; doi:10.1371/journal.pbio.1002233)

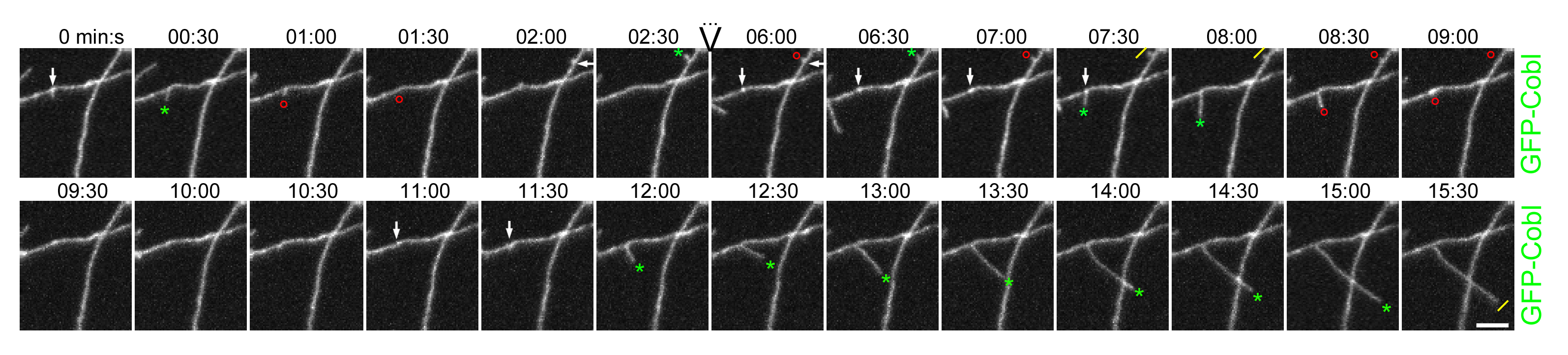

Supplement: S1 Fig — Original maximum intensity projections (MIPs) of the 3-D-time-lapse recording shown as heat map representations in Fig 1C. GFP-Cobl is dynamically enriched at distinct sites within dendrites in immature neurons undergoing dendritogenesis. Initiation of dynamic, dendritic protrusions (marked by green *) often is preceded by Cobl accumulation (white arrows). Retraction events are marked by red ° and static phases with yellow I. Dendrite branch induction is a dynamic process with often several protrusive attempts until a dendritic branch is firmly established and strongly elongated. Three consecutive initiations of a protrusion from the same site are shown. Bar, 5 μm. Please also see S1 Movie. (TIF) [file pbio.1002233.s002.tif]

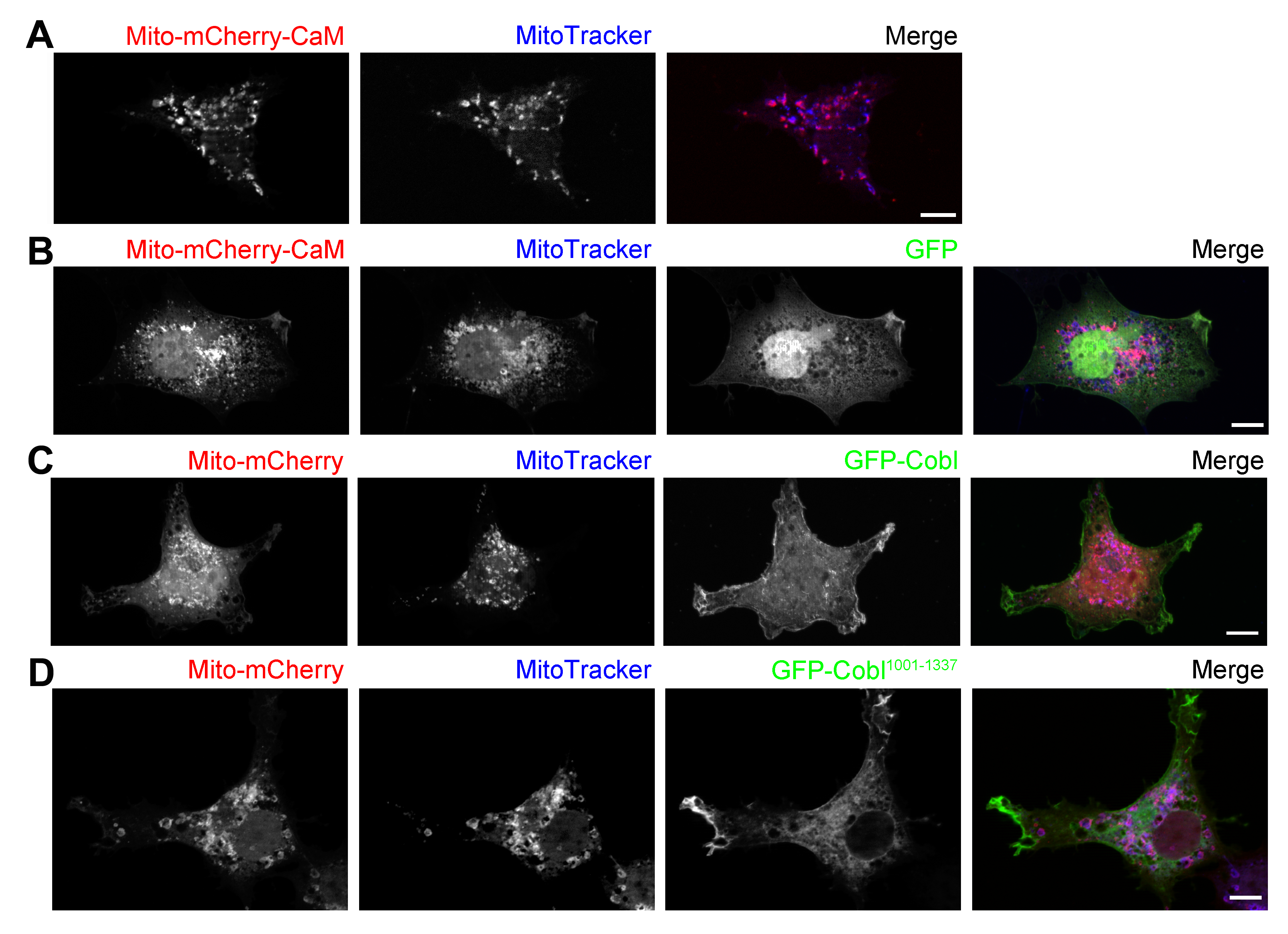

Supplement: S2 Fig — (A) COS-7 cells transfected with Mito-mCherry-CaM show a successful targeting of Mito-mCherry-CaM to mitochondria stained with MitoTracker. (B) Negative control demonstrating that GFP is not recruited to CaM-coated mitochondria. (C,D) Specificity control experiments showing that a related mitochondrially targeted fluorescent protein lacking CaM (Mito-mCherry) is unable to recruit Cobl proteins. Bars in A–D, 10 μm. (TIF) [file pbio.1002233.s003.tif]

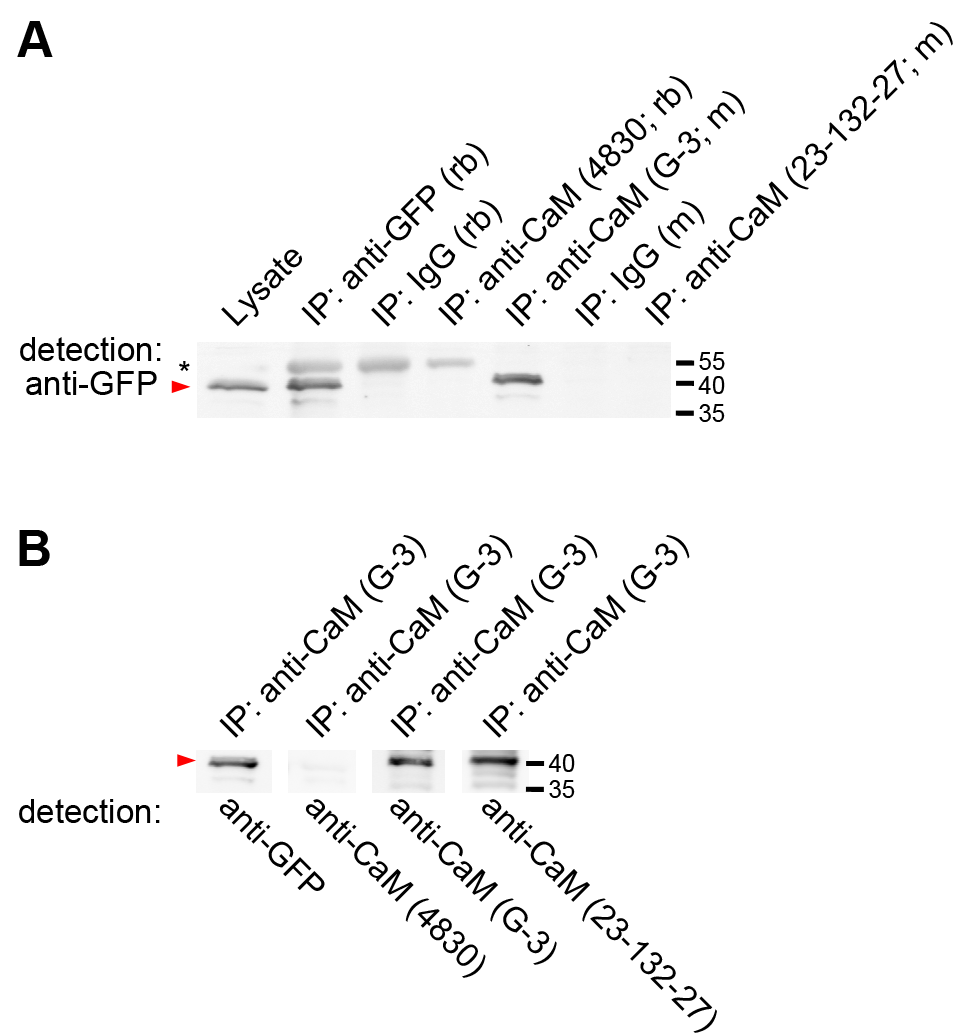

Supplement: S3 Fig — (A) Immunoprecipitation of rat GFP-CaM with anti-GFP antibodies and three commercial anti-CaM antibodies, respectively. Immunoprecipitated material was detected by immunoblotting with anti-GFP antibodies and specificities of immunoprecipitations were controlled for by using IgGs of the respective species. Note that only anti-GFP (positive control) and anti-CaM G-3 antibodies were able to immunoprecipitate GFP-CaM. (B) Immunoblotting analyses of immunoprecipitated GFP-CaM with the different antibodies. Besides anti-GFP antibodies (positive control), also anti-CaM G3 and anti-CaM 23-132-27 antibodies were able to recognize GFP-CaM. (TIF) [file pbio.1002233.s004.tif]

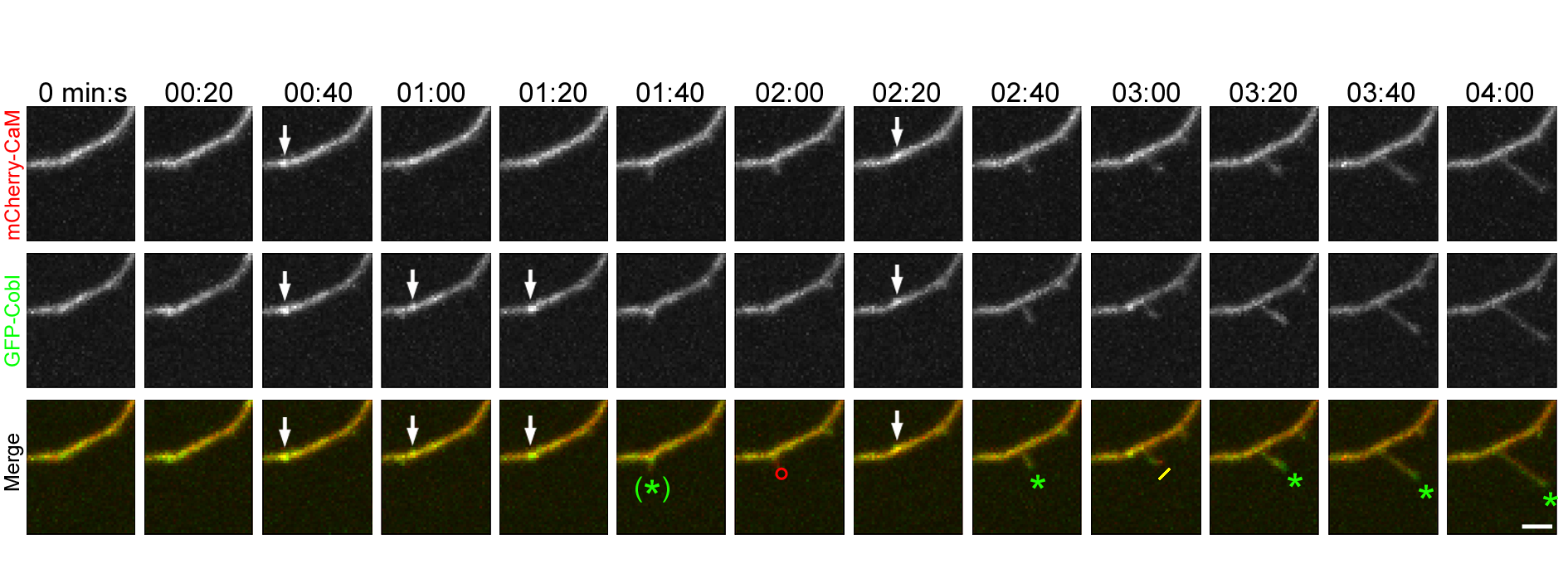

Supplement: S4 Fig — MIPs from 3-D-time-lapse recordings of GFP-Cobl and mCherry-CaM in a dendrite of a primary hippocampal neuron transfected at DIV6 and imaged at DIV7 show that episodes of Cobl accumulation at distinct dendritic sites as well as the induction of protrusions from such sites are accompanied by accumulations of CaM at the same sites. For heat map representation of the individual channels showing GFP-Cobl and mCherry-CaM see Fig 3A. Bar, 2 μm. Please also see S3 Movie. (TIF) [file pbio.1002233.s005.tif]

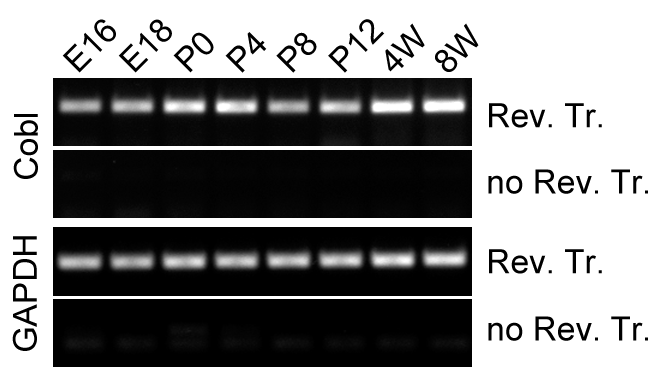

Supplement: S5 Fig — Images of agarose gels (inverted) with amplifications of the Cobl cDNA. GAPDH served as positive control. Reactions without reverse transcriptase (no Rev. Tr.) served as negative controls. (TIF) [file pbio.1002233.s006.tif]

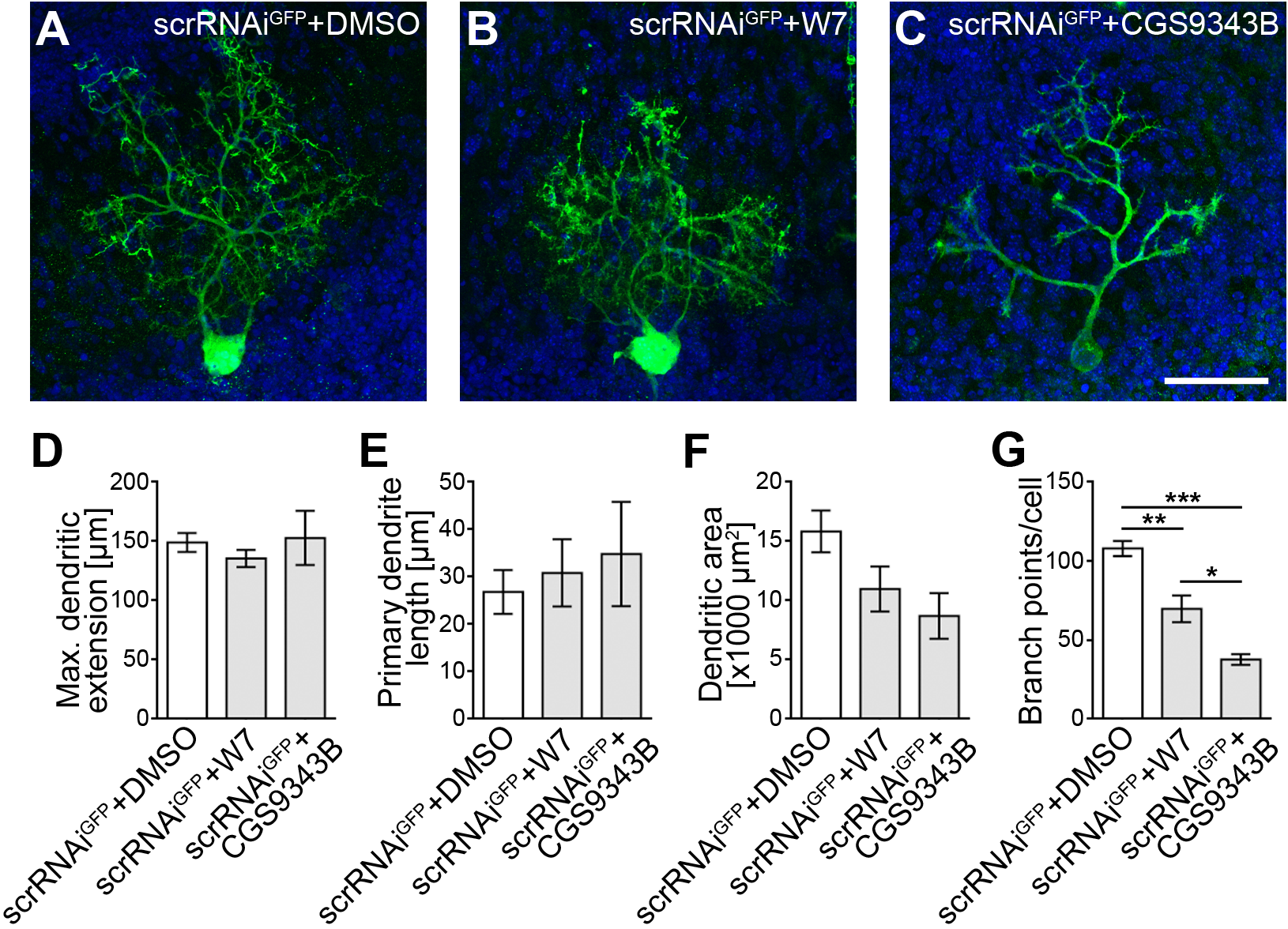

Supplement: S6 Fig — (A–C) Parasagittal cerebellar slices (250 μm) of postnatal day 10 (P10) mice cultured for 2 d showing individual Purkinje cells transfected with a GFP-expressing reporter plasmid and incubated with the indicated CaM inhibitors W7 and CGS9343B as well as with DMSO (solvent control), respectively. DAPI in Blue. Bar, 20 μm. (D–G) Quantification of morphometric parameters of Purkinje cell arborization in cerebellar slice cultures. Data are mean ± SEM. DMSO, n = 6; W7, n = 5; CGS9343B, n = 7 cells. For data underlying D–G, see S1 Data. Statistical significances were tested using one-way ANOVA with Tukey’s post-test. *p < 0.05, **p < 0.01, ***p < 0.001. (TIF) [file pbio.1002233.s007.tif]

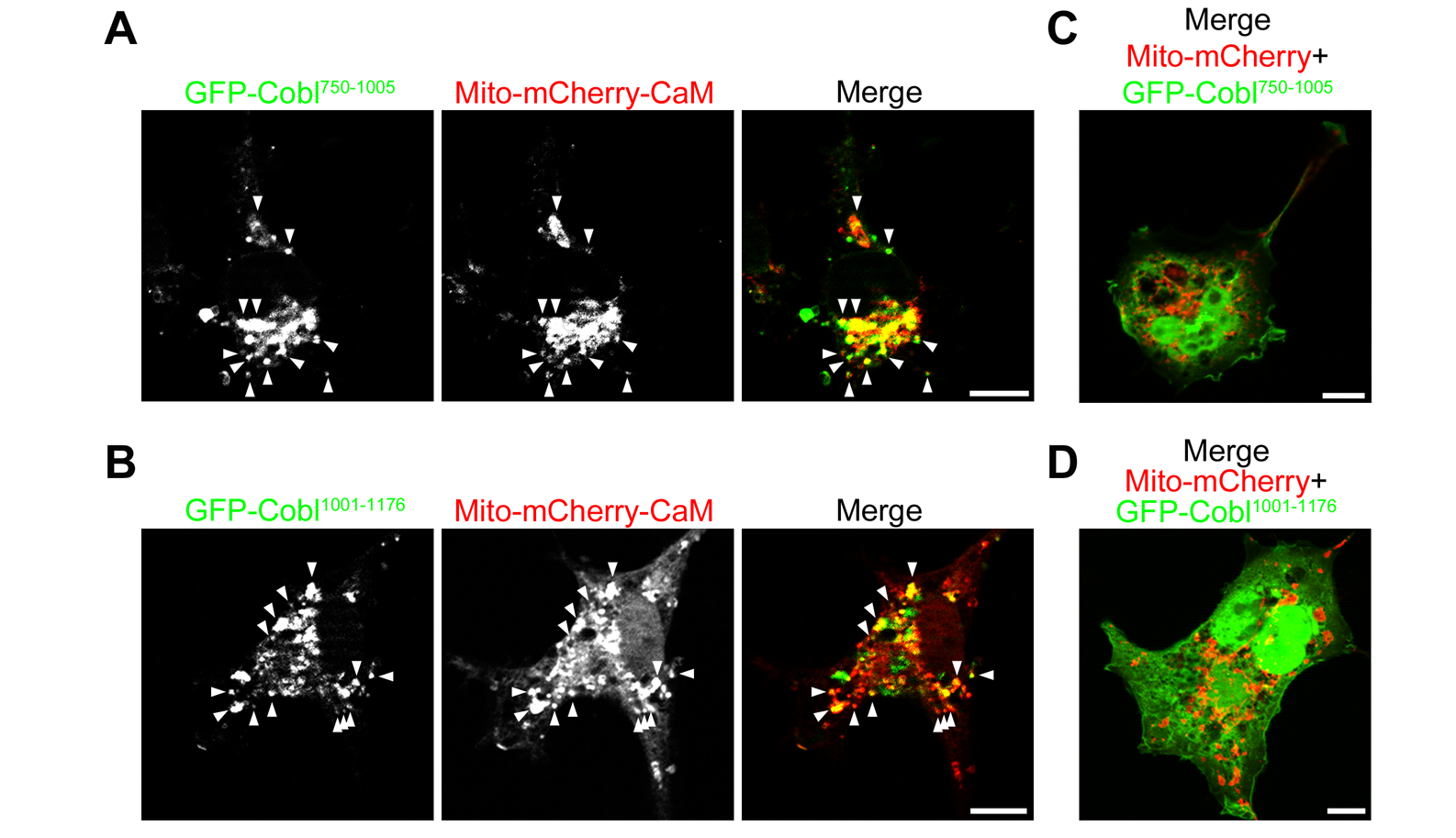

Supplement: S7 Fig — (A–D) Visualization of the Cobl/CaM interaction in intact COS-7 cells by recruitment of GFP-Cobl deletion mutants (Cobl750-1005 and Cobl1001-1176) to mitochondrially anchored mCherry-CaM (Mito-mCherry-CaM) (A,B) but not to Mito-mCherry (C,D). Bars, 10 μm. For further controls see the Supporting Information (S2 Fig). (TIF) [file pbio.1002233.s008.tif]

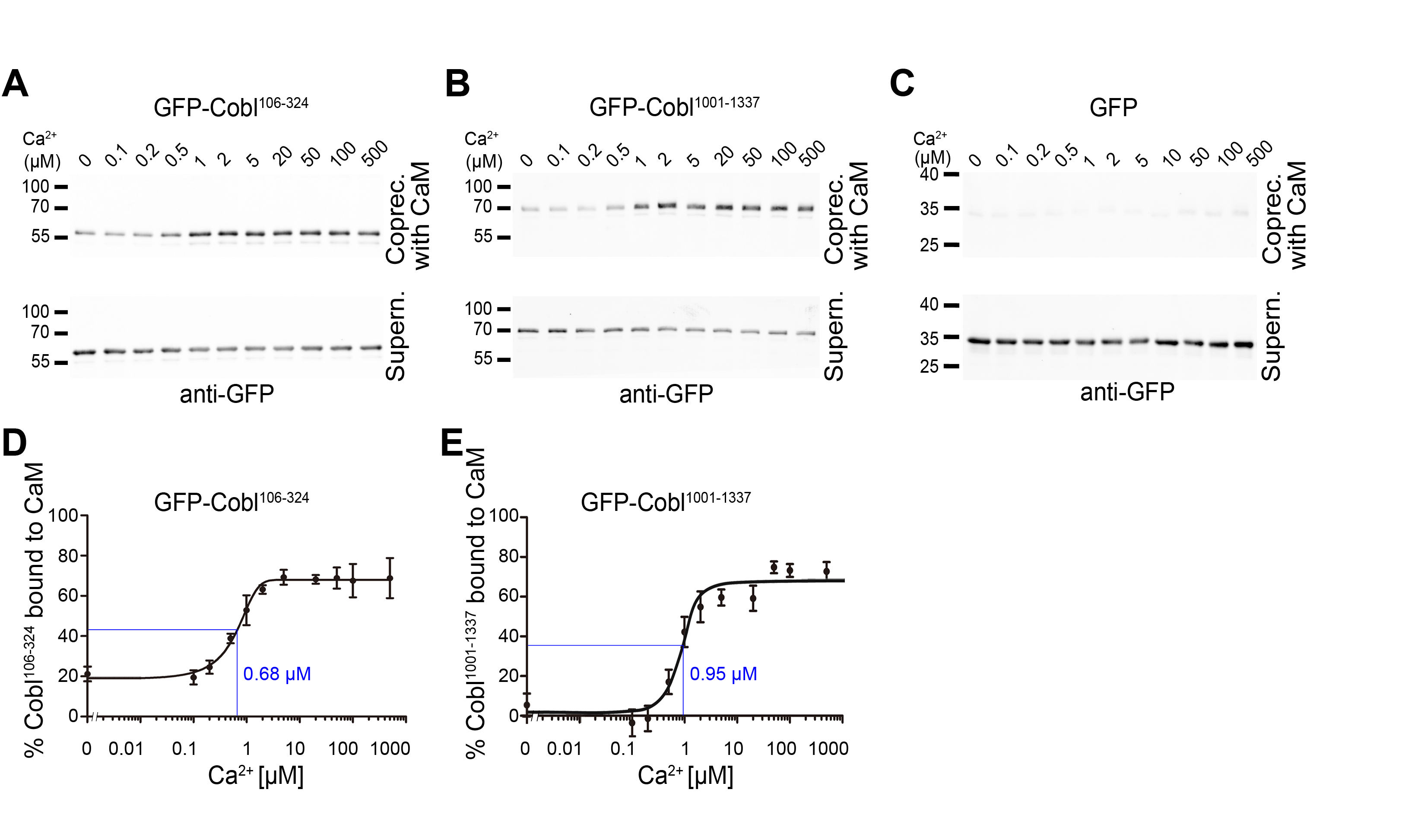

Supplement: S8 Fig — (A–E) Quantitative coprecipitation analyses of GFP-Cobl deletion mutants containing the CaM binding sites within the N-terminal Cobl Homology domain (Cobl106–324) (A) and the CaM binding site located close to the C-terminal WH2 domains (Cobl1001–1337) (B), as well as of a GFP control (C) with immobilized CaM under different calcium concentrations. Note that both Cobl fusion proteins bind to CaM in a specific manner (A–C) and that quantitative Western blotting analyses (D,E) show that half-maximal binding is already reached at 0.68 μM and 0.95 μM Ca2+, respectively. Please also note that 2 μM Ca2+ used in some biochemical assays of this study corresponds to about 80%–90% of maximal binding observed and that 500 μM Ca2+ ensures plateau levels of CaM association. GFP-Cobl106–324, n = 4; GFP-Cobl1001–1337, n = 7; GFP, n = 3. For data underlying D and E see S1 Data. (TIF) [file pbio.1002233.s009.tif]

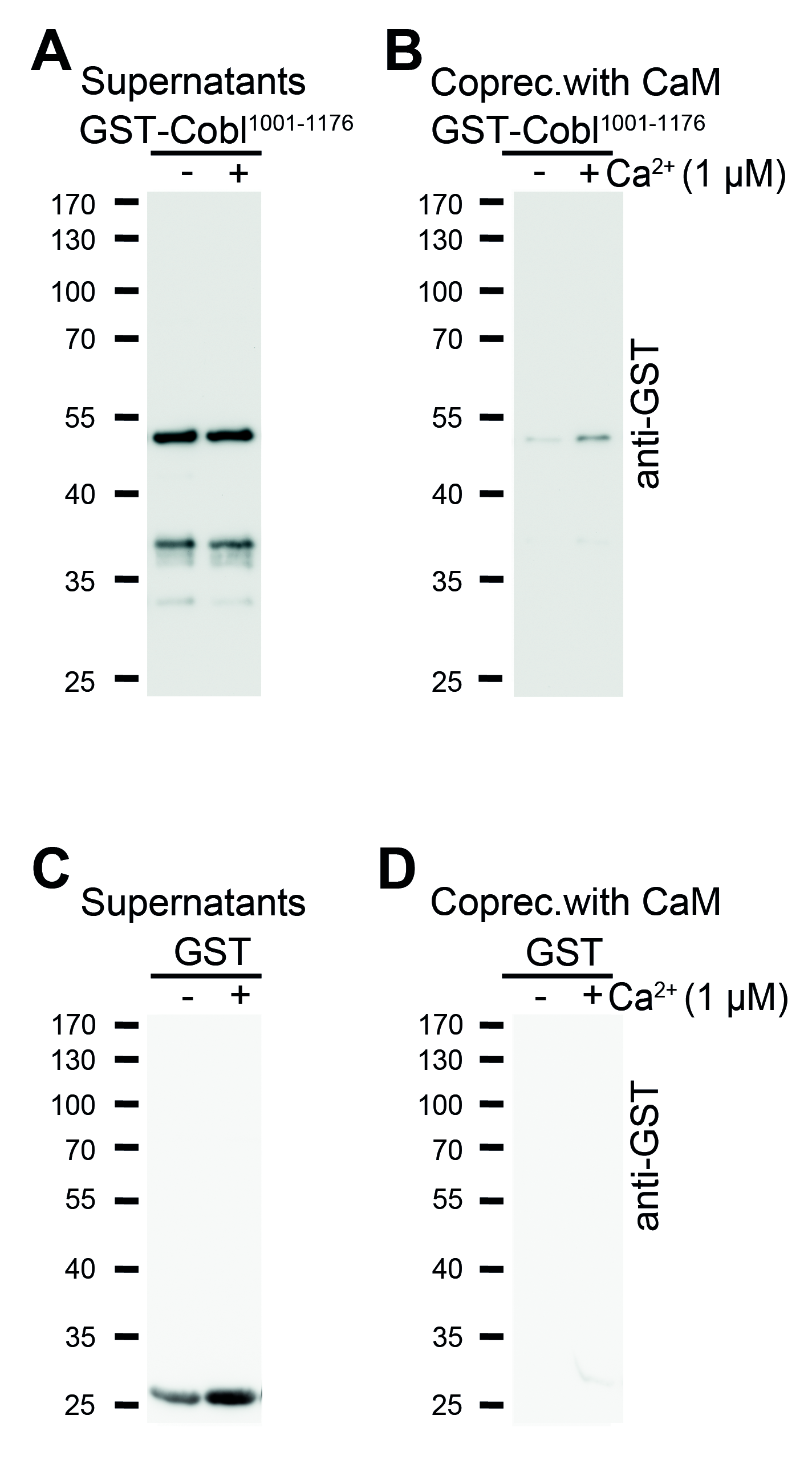

Supplement: S9 Fig — (A–D) In vitro reconstitution experiments using immobilized CaM and purified GST-Cobl1001-1176 (A,B) and a GST control, respectively (C,D), show that the Cobl/CaM interaction is direct and Ca2+-dependent. (TIF) [file pbio.1002233.s010.tif]

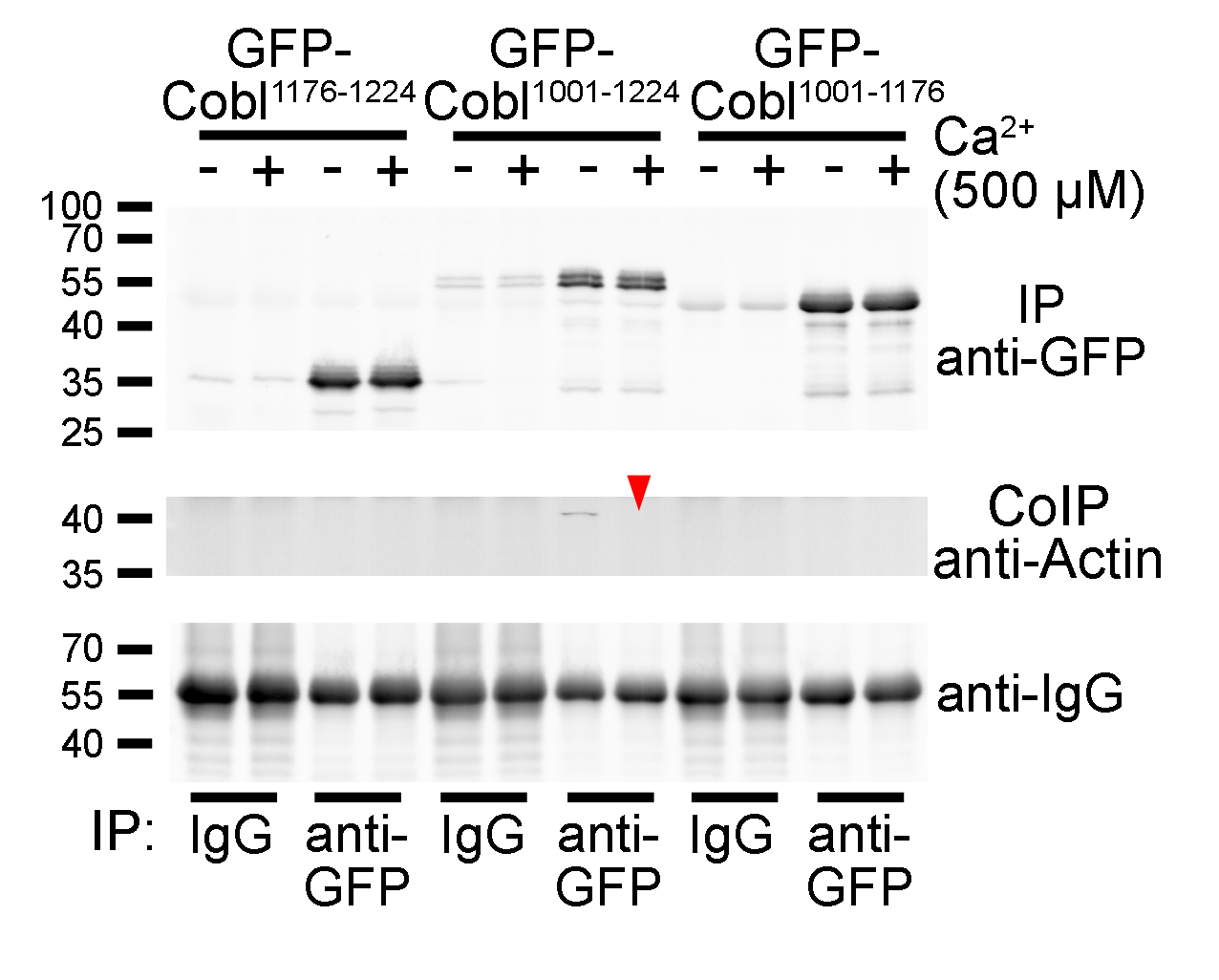

Supplement: S10 Fig — Immunoblottings of immunoprecipitations of GFP-Cobl1176-1224, GFP-Cobl1001–1224, and GFP-Cobl1001-1176 with anti-GFP antibodies from HEK293 lysates (IP, anti-GFP) show that specific coimmunoprecipitation of endogenous actin (CoIP, anti-actin) requires a combination of the first WH2 domain and the CaM binding interface and demonstrate that this actin association of the first WH2 domain of Cobl is inhibited by Ca2+ addition (red arrowhead). (TIF) [file pbio.1002233.s011.tif]

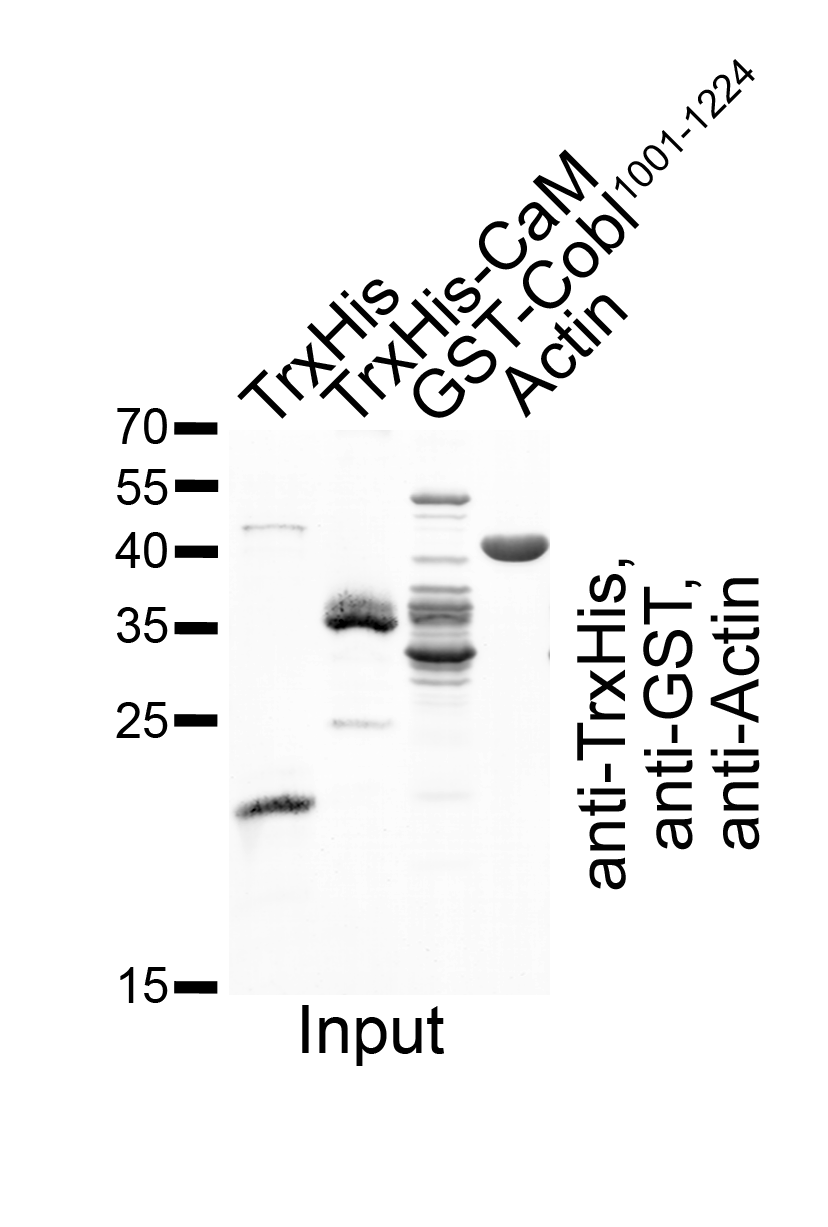

Supplement: S11 Fig — Immunoblot analysis of the input of purified proteins used for the in vitro reconstitution of the Ca2+/CaM-mediated suppression of the actin binding of the first WH2 domain of Cobl (see Fig 5I) by a mix of anti-TrxHis, anti-GST, and anti-actin antibodies. (TIF) [file pbio.1002233.s012.tif]

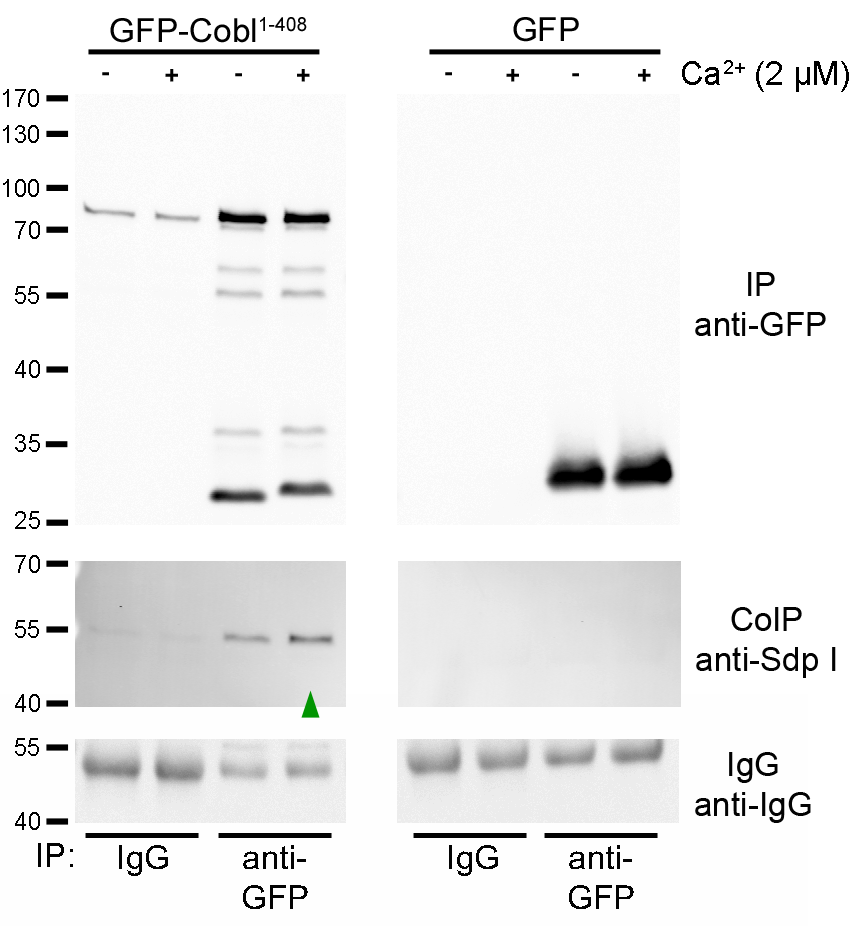

Supplement: S12 Fig — Coimmunoprecipitations of GFP-Cobl1-408/Flag-syndapin I (Flag-Sdp I) along with a corresponding GFP control from HEK293 cells under Ca2+-free conditions (−) and 2 μM Ca2+ (+). Note that the syndapin I interaction with Cobl is promoted upon increasing the Ca2+ concentration (indicated by the green upright arrowhead). The data shown corroborate the quantitative coimmunoprecipitation analyses shown in Fig 8A–8C under increased salt conditions (100 mM NaCl). (TIF) [file pbio.1002233.s013.tif]

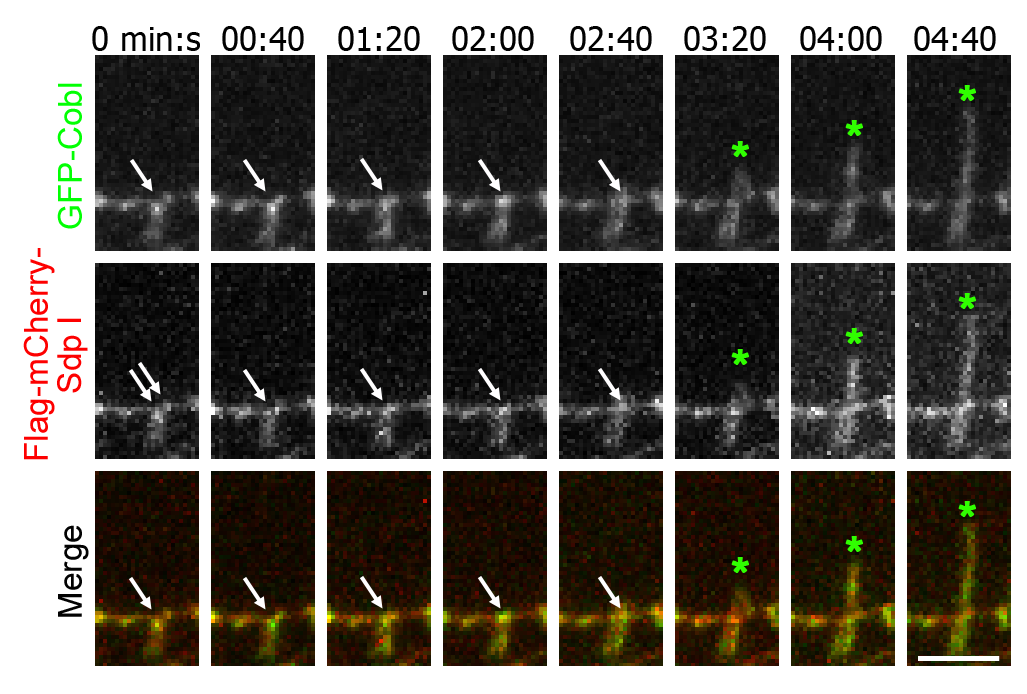

Supplement: S13 Fig — Time-lapse spinning disc microscopy images (MIPs) of a dendritic protrusion (*) emanating from a Cobl and syndapin I-enriched site (arrows). For heat map representations of the data for GFP-Cobl and Flag-mCherry-syndapin I, see Fig 9B. Bar, 5 μm. (TIF) [file pbio.1002233.s014.tif]

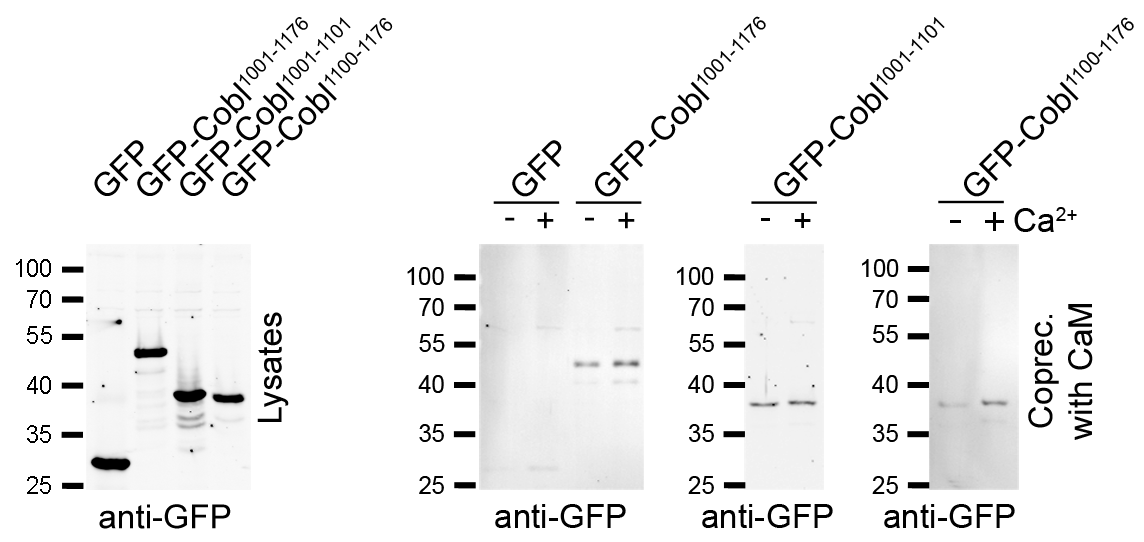

Supplement: S14 Fig — Coprecipitation experiments with immobilized CaM and GFP-Cobl deletion mutants in presence and absence of 500 μM Ca2+ identified two independent sites within Cobl1001-1176 that specifically associated with CaM. GFP fusion proteins expressed (lysates) and coprecipitated with CaM, respectively, were detected by immunoblotting with anti-GFP antibodies. (TIF) [file pbio.1002233.s015.tif]

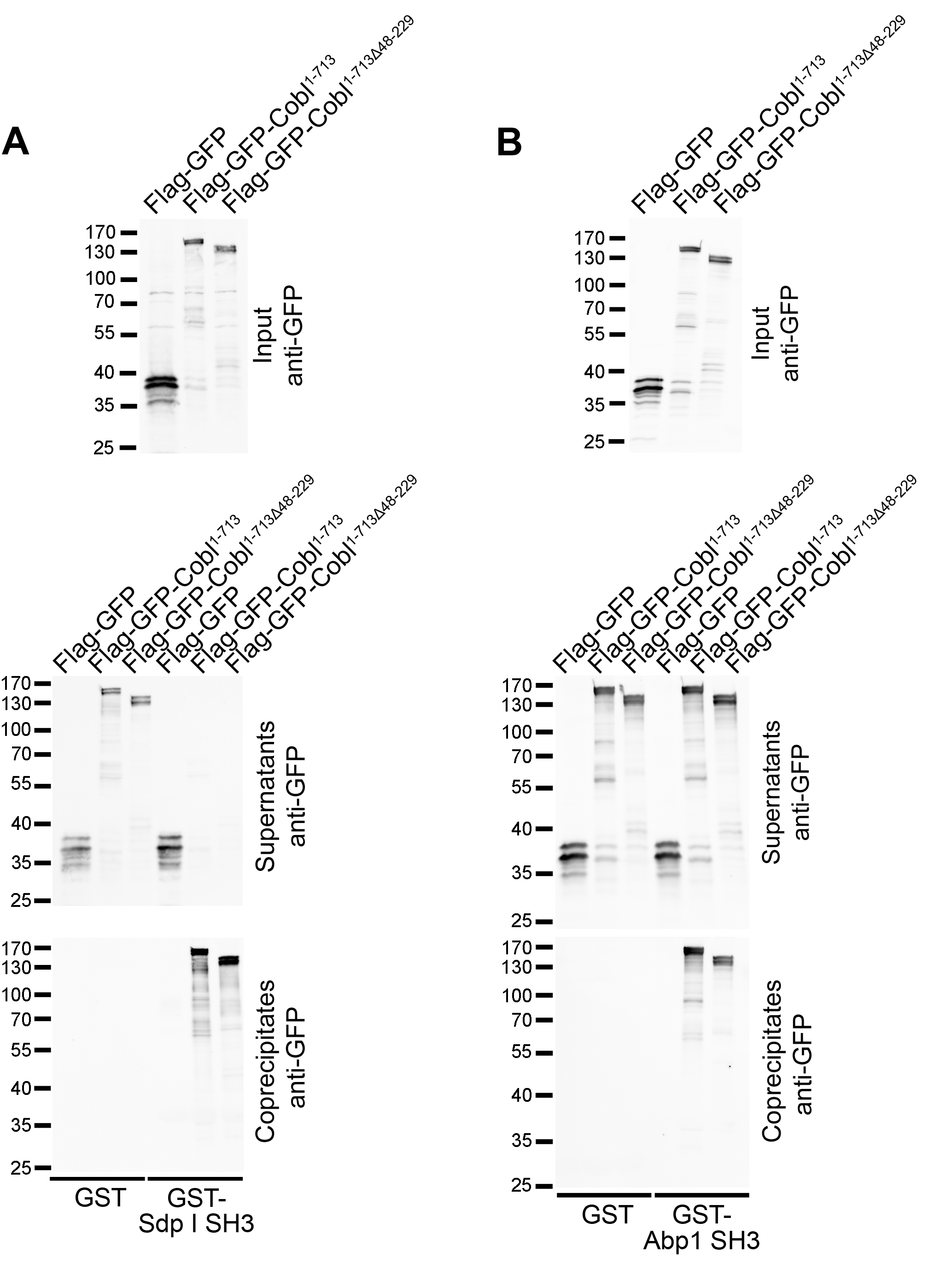

Supplement: S15 Fig — (A,B) Coprecipitation experiments demonstrating that both Flag-GFP-Cobl1-713 and the corresponding CaM binding-deficient mutant Flag-GFP-Cobl1-713∆48–229 specifically associate with immobilized GST-SH3 domain fusion proteins of two components crucial for Cobl’s functions in dendritogenesis, syndapin I (A) and Abp1 (B). (TIF) [file pbio.1002233.s016.tif]
